# Neural decision dynamics underlying reinforcement learning and working memory

**DOI:** 10.1016/j.isci.2026.115471

**Published:** 2026-03-27

**Authors:** Mads L. Pedersen, Erik R. Frogner, Lars T. Westlye, Torgeir Moberget

**Affiliations:** 1 Department of Psychology, University of Oslo, Oslo, Norway; 2 Center for Precision Psychiatry, Division of Mental Health and Addiction, Oslo University Hospital, Oslo, Norway; 3 KG Jebsen Centre for Neurodevelopmental Disorders, University of Oslo, Oslo, Norway; 4 Department of Psychology, Pedagogy and Law, Kristiania University of Applied Sciences, Oslo, Norway

**Keywords:** biological sciences, clinical neuroscience, natural sciences, neuroscience

## Abstract

Learning relies on multiple cognitive mechanisms—including reinforcement learning (RL) and working memory (WM)—forming internal value representations guiding choice. However, it remains unclear how these values are transformed into choices, and how this transformation relates to RL and WM. We analyzed electroencephalography (EEG) data from 510 participants performing the RLWM task. An RLWM-linear ballistic accumulator (RLWM-LBA) model was applied, linking RL- and WM-derived policy estimates to evidence-accumulation dynamics. With model-derived event-related potential (ERP) analyses, we tested whether neural signatures of RL and WM persist when accounting for choice dynamics, and whether a neural evidence accumulation signal—the centro-parietal positivity (CPP)—emerges in a learning context. Our findings replicate distinct neural correlates for RL and WM and reveal a CPP signal reflecting uncertainty in learned value representations. CPP signals improve model fit and are differentially linked to RL and WM across cognitive load, supporting their role in shaping learning-related decision dynamics.

## Highlights

• EEG reveals accumulation dynamics during reinforcement learning decisions
• CPP signal tracks trial-by-trial evidence from latent value estimates
• Reinforcement learning and working memory differentially shape CPP
• Neural CPP signals improve computational model fit

## Graphical abstract

**Figure:**
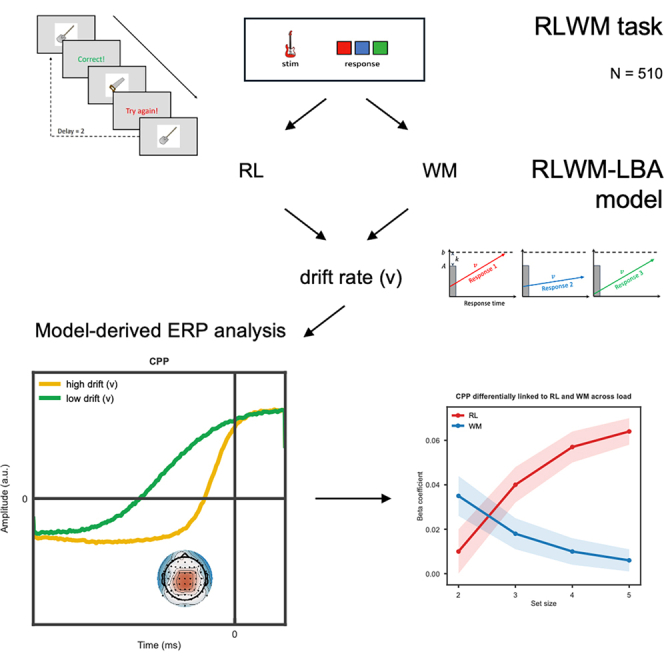

## Introduction

The temporal dynamics of decision-making in learning contexts have been studied by modeling how reinforcement-based internal value representations are transformed into choices and response time distributions.^1^^,^^2^^,^^3^^,^^4^^,^^5^ Sequential sampling models, including the drift diffusion model (DDM) and linear ballistic accumulator (LBA) model, have been central to these efforts by capturing decision-making as the accumulation of evidence until a decision threshold is reached.^6^^,^^7^^,^^8^

Much of what we currently know about the underlying neural dynamics of decision-making has emerged from studies of perceptual decisions.^9^^,^^10^^,^^11^ A candidate neural signature that builds gradually until a decision boundary is crossed, has been identified across species, including humans and non-human primates, and across recording techniques, including single-cell recording, functional magnetic resonance imaging (fMRI) and electroencephalography (EEG).^12^^,^^13^^,^^14^ EEG recordings in humans have identified the centro-parietal positivity (CPP) as a candidate event-related component reflecting a neural instantiation of evidence accumulation.^15^^,^^16^^,^^17^^,^^18^ Despite advances in perceptual and preference-based decision-making^19^ (but see Frömer et al.^20^), the neural dynamics of evidence accumulation are largely unexplored in learning contexts, where decisions are guided by feedback-driven internal value estimates or action policies. Specifically, it remains unknown whether the neural decision-making processes in instrumental learning mirror the neural accumulation dynamics observed in perceptual and preference-based tasks.

Parallel research has emphasized that instrumental learning, even when limited to simple stimulus-response associations, involves multiple cognitive processes rather than a single reinforcement learning (RL) mechanism.^21^^,^^22^^,^^23^ The reinforcement learning-working memory (RLWM) framework has shown that working memory (WM) and RL differentially contribute to learning stimulus-response associations.^21^^,^^24^ Importantly, the RLWM framework has demonstrated that the balance and cooperation between RL and WM vary systematically with cognitive demands such as load and delay, highlighting the context-dependent dynamic interplay between these cognitive systems.

Recently, these lines of research converged in the development of the RLWM-LBA model.^25^ The RLWM-LBA model combined the RLWM approach with the LBA model,^6^ linking learning processes to the temporal dynamics of decision-making. Using the RLWM-LBA model in combination with EEG, we can now address two central questions: first, can neural correlates of evidence accumulation be identified in learning contexts? Second, how do RL and WM influence this neural accumulation process?

In the present study including data from a large (*N* = 510) convenience sample of healthy volunteers aged 11 to 43 years, we utilize a combined computational modeling and EEG approach to investigate these questions. We first replicate and extend previous EEG results for RL and WM processes fit to choices (without RTs),^24^^,^^26^ confirming distinct temporal and topographical neural signatures. Next, by analyzing EEG signals in conjunction with model-derived drift rates, representing the speed of evidence accumulation, we identify a CPP signal reflecting key characteristics of neural evidence accumulation.

Moreover, we find that incorporating a model-naïve, trial-by-trial measure of CPP buildup rate to inform drift rate improves the RLWM-LBA model’s fit to choice and RT, providing support to the assumptions that the CPP reflects the rate of evidence accumulation. Finally, our analyses reveal that the relative contributions of RL and WM to the neural accumulation process shift systematically with cognitive load: the influence of RL increases while the influence of WM decreases as the number of stimulus-response associations to be learned within a block increases.

In summary, we here identify neural correlates of evidence accumulation in an instrumental learning task and insights into the neural processes of how RL and WM interact to guide choice behavior during learning.

## Results

**Figure 1:**
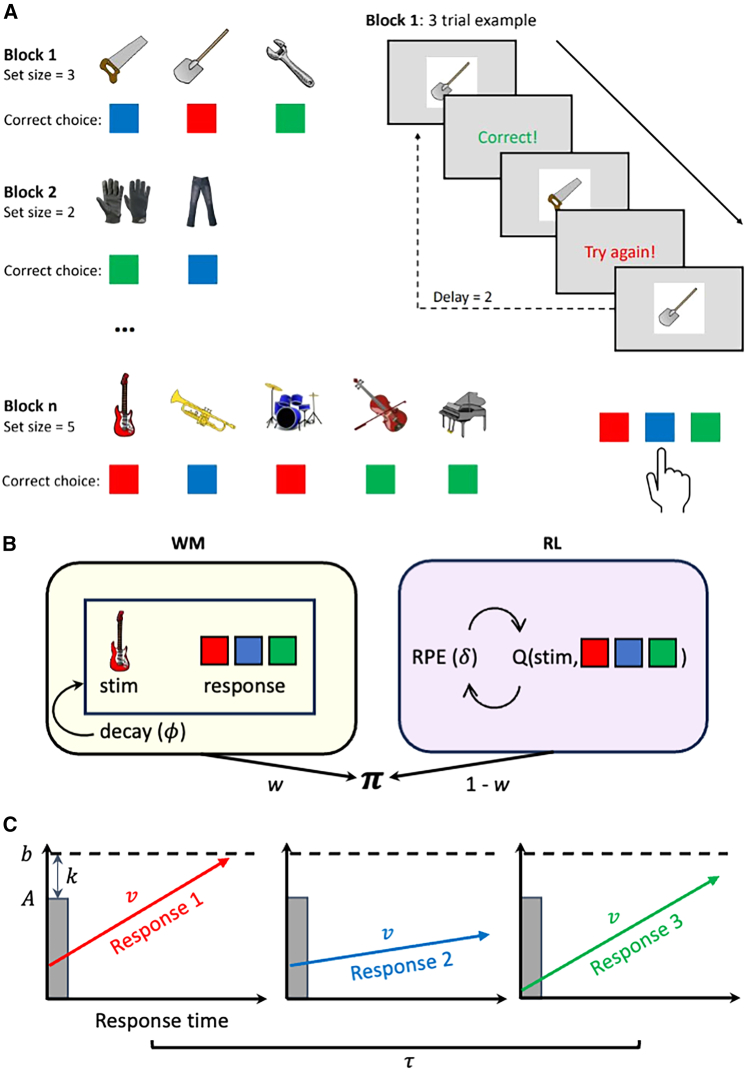
Experimental task and RLWM-LBA model (A) Illustration of RLWM task. Participants learned to associate stimuli with keypresses (here represented as colors) through deterministic feedback. The number of stimuli to learn varied across blocks, thereby challenging WM capacity. Adapted from Master et al.^27^ (B) Graphical illustration of the interaction of RL and WM in the RLWM model. The RLWM model assumes the WM module directly learns the stimulus-response association, but is subject to decay, while the RL module learns by updating values through reward prediction errors. The action policy π is a combination of value estimates from a proportion of the WM and RL modules, where the proportion *w* varies by set size depending on the propensity to use WM and WM capacity. Adapted from McDougle and Collins.^25^ (C) Graphical illustration of the LBA model. The model assumes a race between accumulators, where the winning response and response time is defined by the accumulator first crossing the decision threshold *b*. Importantly, the RLWM-LBA model assumes the drift rate (*v*) for each response is defined by the action policy pi. Adapted from Brown and Heathcote.^6^ RLWM, reinforcement learning working memory; LBA, linear ballistic accumulator.

Five hundred sixty-five participants performed the RLWM task, a stimulus-response association task where subjects learn to associate stimuli with one of three button presses (Figure 1A). The RLWM task was designed to tax the capacity and decay of the WM system by manipulating the number of stimuli (2, 3, 4, or 5) to be learned within a block and by varying the delay with which the same stimulus was presented again. EEG data were recorded while participants performed the task to assess neural correlates of WM and RL and their impact on choice dynamics. Data from 55 participants were excluded because the EEG data quality was deemed poor and/or less than 50% of original trials remained after removal of erroneous or noisy trials, resulting in a final sample of 510 participants (see STAR Methods for details).

### Behavioral results

**Figure 2:**
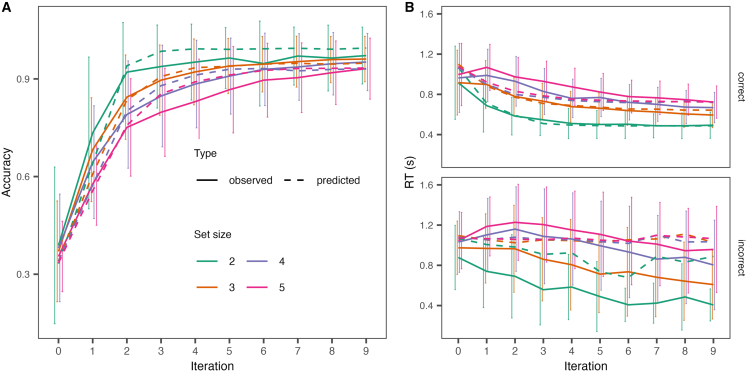
Model fit Observed and predicted results for (A) accuracy and (B) response time (RT) for correct and incorrect choices across set size. The *x* axis shows the number of successive stimulus presentations. The model captures the increase in accuracy and reduction in RT with learning, and further that these effects are stronger in low compared to high set sizes. Error bars = standard deviation.

Observed measures of performance on the RLWM task showed signs of involvement of both RL and WM. First, accuracy improved, and RT decreased with stimulus presentations (Figure 2), indicative of incremental learning as reflected in RL. Second, accuracy improved, and RT decreased more quickly in lower set sizes (Figure 2), indicative of the use of a WM system which successfully kept stimulus-response associations active in lower set sizes but were challenged by capacity-limitations and decay for higher set sizes. Further corroborating evidence of the involvement of both RL and WM can be seen in how accuracy was influenced by set size, number of stimulus presentations and delay (Figure S1). Lastly, we observed lower overall accuracy (mean difference = −0.02, 95% HDI = −0.02, −0.01) and slightly faster reaction time (mean difference = −0.02, 95% HDI = −0.04, 0.00) in the youth cohort compared to the pregnancy cohort.

### Computational model

#### Model fit

Trial-by-trial choice and RT data from the RLWM task was analyzed with a hierarchical Bayesian version of the RLWM-LBA model^25^ (Figures 1B and 1C; see distribution of parameter estimates in Figures S2 and S3). The model was shown to provide good fits to data, capturing the increased accuracy and decreased RT with stimulus iteration, and how these effects varied across set size (Figure 2).

#### Latent variables

Our main interest in the RLWM-LBA model here was to investigate neural correlates of learning and decision-making. To do so, we extracted model-inferred latent trial-by-trial variables of stimulus-response associations (*Q* values) for both the RL and WM system and their combination into drift rates, reflecting the speed of evidence accumulation. *Q* values for the RL system were updated after feedback with the reward prediction error, scaled by learning rate. *Q* values for the WM-system were updated perfectly (i.e., learning rate = 1), but values were drawn toward initial values after each trial to reflect memory decay or interference from subsequent trials updating in WM. The contribution of *Q* values from the two systems onto choice and RT depended on estimated individual parameters of propensity to use WM vs. RL, *ρ*, and by WM capacity *C* (see STAR Methods for details). Trial-by-trial drift rates were used as predictors in the EEG analysis as a measure of relative uncertainty in stimulus-response associations, akin to choice difficulty in perceptual decision-making tasks.

#### EEG results

Our large cohort combined with the use of the RLWM-LBA model provided a platform to build upon prior research exploring the neural underpinnings of RL, WM, and choice dynamics.

First, we sought to replicate established EEG correlates of RL and WM using the RLWM-LBA model, which also accounts for choice RT distributions. This served as a validation step to assess the stability of previously reported neural signatures under a different modeling framework.

Second, we leveraged the detailed trial-level drift rate predictions from the RLWM-LBA to test for neural evidence accumulation signals during instrumental learning—specifically, a CPP-like component analogous to that found in perceptual decision-making.^11^

Additionally, as stimulus and response potentials can overlap in free-RT tasks, we applied the unfold toolbox as a control analysis throughout^28^ to verify that effects of RL, WM, and drift rate modulation remain after deconvolution of ERP signals linked to processing of the preceding stimulus.^20^

#### Replicating EEG correlates of RL and WM during learning

Previous studies have used EEG to investigate the scalp topographies and latencies of electrophysiological brain signals related to trial-by-trial variability in RL and WM parameters.^24^^,^^26^ In these analyses, EEG voltages at all electrodes and time points were fitted by general linear models, where the EEG signal is modeled as the sum of a set of independent variables/predictors, plus random noise. Specifically, they investigated the effects of set size, delay, and the stimulus-response value in the RL system for the chosen option.

**Figure 3:**
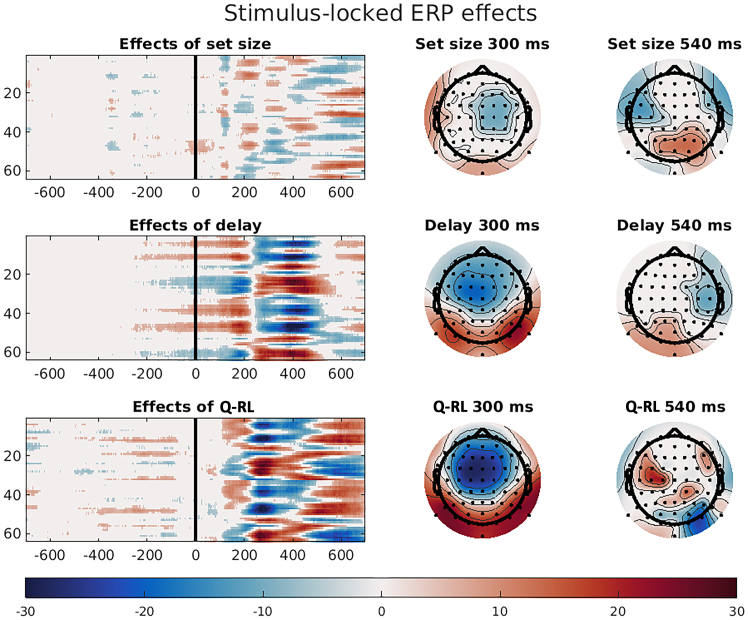
Stimulus-locked ERP effects of RL and WM predictors Colored tiles in the left images denote significant *t* values across time points (*x* axis) and electrodes (*y* axis). Right images show scalp maps of effects of set size, delay and Q-RL values at early (300 ms) and late (540 ms) after stimulus onset. Colored electrodes and time points show significant negative (blue) or positive (red) *t* values (Bonferroni corrected across electrodes, time points, and estimated model parameters), with the scale ranging from −30 to 30.

Consistent with earlier studies,^24^^,^^26^ we found a prominent frontal negativity around 300 ms after stimulus-onset for *Q* values, as well as a similar pattern at the same time point for delay (Figure 3). We also observed the previously described parietal positivity around 540 ms for effects of set size. However, unlike these previous reports, we additionally observed an early frontal negativity for set size.

Similar to Rac-Lubashevsky et al.,^26^ we found a late temporal component of Q-RL values (Figure 3). Also consistent with previous studies, we found significant early frontal and parietal effects of delay, which was assumed to reflect the reduced utility and activation of the WM system for longer delays, when it is likely that the action value has decayed from the WM system.

Next, we used the estimated beta-parameters from the linear models to reconstruct ERPs related to specific variables after removing effects of other modeled variables (i.e., “corrected ERPs”). Figure S4 shows these corrected ERPs demonstrating effects of set size, delay, and Q-RL on electrodes FCz and CPz. As can be seen, for set size and delay, there was a marked difference between the easiest conditions (set size 2 and delay 1) and the rest, while Q-RL showed a more continuous effect across the four quartiles.

Given the systematic response time effects associated with all modeled variables, all main ERP-effects remained when deconvolving potentials associated with stimulus and response using the unfold^28^ method (Figures S5 and S6). These control analyses suggest that the main reported effects are not an artifact of overlapping activity from stimulus and response potentials.^20^

#### EEG reveals a CPP signal during learning that reflects latent RL, WM, and drift rate dynamics

Our second aim was to search for a neural evidence accumulation signal in stimulus-response learning akin to the CPP component identified in perceptual decision-making.^11^^,^^29^^,^^30^ Several assumptions should be met for a neural signal to be deemed a potential evidence accumulation signal. First, activity should rise until a threshold is reached, and this threshold should be close in time to the motor response. Further, there should be an effect of choice difficulty, in which activity should have an earlier onset and accumulate more slowly for more difficult and more prolonged decisions. In addition, several studies have found an effect of difficulty in peak amplitude, in which easier decisions peak at a higher threshold prior to motor response.^16^^,^^31^^,^^32^ Lastly, it has recently been argued that CPP signals may arise artifactually from the overlapping activity from stimulus and response potentials,^20^ but see O’Connell et al.^33^ To account for such effects, we ran additional analyses using the unfold method^28^ to disentangle activity linked to stimulus and response.

As there were strong effects of set size on drift rate, we calculated drift rate *Z* scores and ran analyses separately for each set size. For visualization of drift rate effects, we divided drift rate into quartiles for each set size.

#### Buildup of CPP scales with drift rate

**Figure 4:**
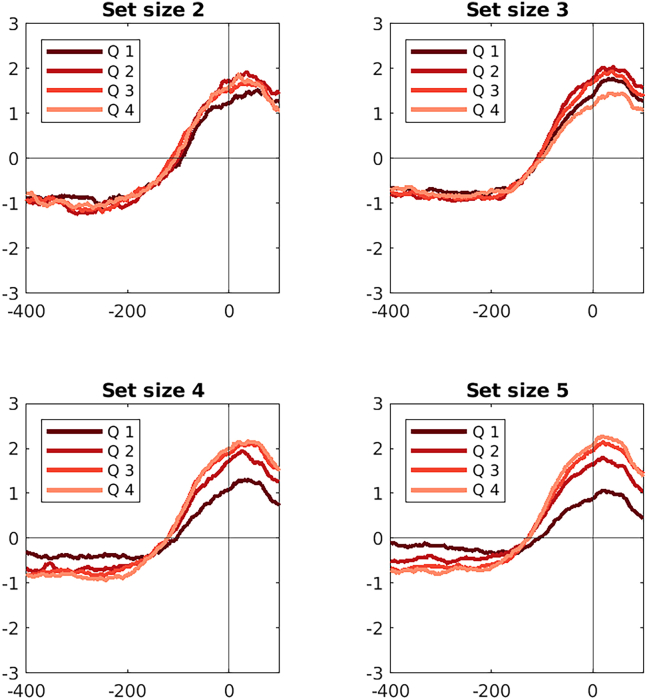
Corrected ERP effects of drift rate on electrode CPz Inferred drift rates were divided into quartiles (*Q*) for each subject and set size condition. Q1 represents the trials with the least and Q4 the trials with the most evidence for the chosen (correct) option. The *x* axis shows time (in ms) relative to the response, while the *y* axis shows ERP amplitudes (in microvolts). Black dots denote significant time points (after Bonferroni-correction across time points, electrodes, and tested variables).

Inspection of the grand average ERPs from electrode CPz showed that this CPP response peaked close to response execution (Figure 4). Specifically for higher set sizes, the CPP response was modulated by drift rate, with prolonged activity for lower drift rates and steeper slope and higher amplitude at response for higher drift rates. Effects of drift rate on CPP were also present when applying the unfold method, although the earlier effect in the −400 to −200 ms time window disappeared (Figures S8 and S9). Corroborating model-inferred effects of drift rate, we also identified an effect of observed behavior in that RT quartiles modulated the CPP response (Figures S10 and S11). These effects were also present when applying the unfold method (Figures S12 and S13), although again the early effects were less pronounced.

Finally, whole-scalp and time-resolved analyses confirmed that these drift-rate effects were most pronounced at higher set sizes and were spatially centered over centro-parietal electrodes (Figure S7), supporting the interpretation of the observed signal as a CPP-like accumulation process during learning.

#### Trial-by-trial slope improves model fit

Next, we set out to investigate whether the buildup observed in the CPz could enhance the RLWM-LBA model’s fit, which would further validate the CPP component as a candidate neural implementation of evidence accumulation.^2^^,^^31^^,^^34^ Specifically, we wanted to test whether a trial-by-trial CPP slope signal, as a proxy for the neural evidence accumulation, could inform the trial-by-trial drift rate and thereby provide information over and above what the model could derive from behavior alone. To derive a CPP slope signal we extracted the amplitudes from the CPz at −200 and −50 ms prior to response on each trial and calculated the slope across these time points. We then extended our baseline RLWM-LBA model by including the CPP slope as a regressor on the drift rate scaling parameter *η*:

$$\eta_{t}\;∼\;\eta +{\mathrm{CPP}}_{\mathrm{slope},t}\times \beta_{\mathrm{slope}}$$

Thus, in the neurally informed model choice probabilities π were scaled with a scaling parameter η that varied from trial-by-trial, dependent on the CPP slope regressor and *β*_*slope*_ coefficient:

$$V_{a,t}=\eta_{t}\left(\frac{\pi_{i,t}}{H_{\mathrm{prior},t}}\right)$$

This neurally informed model significantly improved model fit relative to the baseline model, which assumed a fixed scaling parameter η (elpd_diff: 1609, SE: 48.5), far exceeding its associated uncertainty. Given the scale of the dataset, this corresponds to a consistent per-trial improvement in predictive log likelihood, indicating that the gain reflects widespread improvements in predicting RT and choice rather than a small number of extreme observations. Additionally, the regression coefficient *β*_*slope*_ was positive and reliably different from zero (mean: 0.094, HDI [95%] = 0.077–0.111), such that higher values of trial-by-trial neural indices of evidence accumulation increased the scaling parameter, directly predicting variability in the rate at which learned value estimates translate into decision evidence.

#### WM and RL differentially linked to CPP slope across set size

Finally, we explored whether value estimates from the RL and WM modules differentially relate to the rate of the proposed evidence accumulation process across set size. To do so, we regressed *Q* value estimates of RL and WM, calculated as quartiles within each set size, on the trial-by-trial CPP slope signal:

$${\mathrm{CPP}}_{\mathrm{slope}}\;∼\;{Q-RL}_{\mathrm{quartile}}*\mathrm{set}\;\mathrm{size}+{Q-WM}_{\mathrm{quartile}}*\mathrm{set}\;\mathrm{size}$$

**Figure 5:**
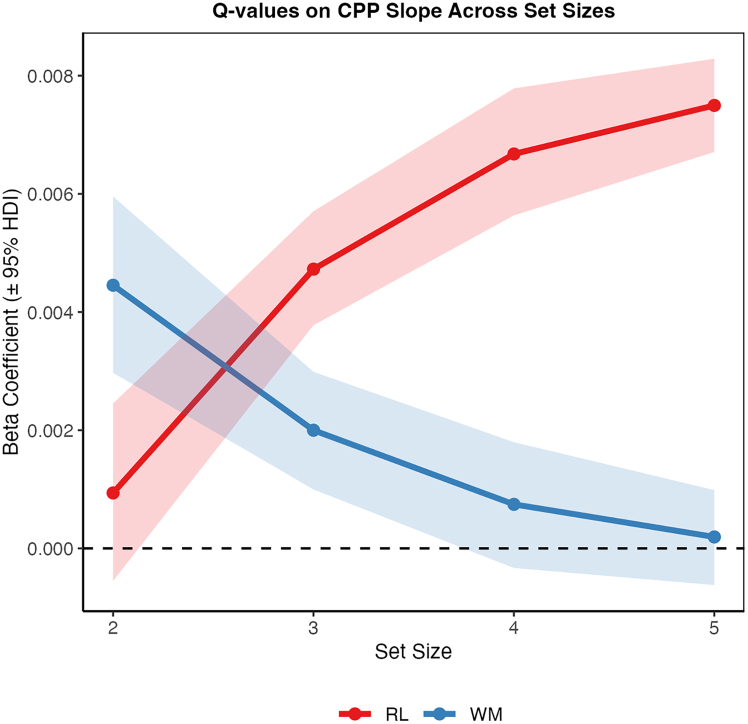
Associations of Q-RL (red) and Q-WM (blue) quartiles on CPP slope across set sizes Beta coefficients with 95% highest density interval (HDI). The associations increased with set size for RL and decreased for WM, in line with assumptions of increased (decreased) influence of RL (WM) on choice with load.

This analysis was used to measure the degree to which variability in value estimates were linked to CPP slope, where a higher beta coefficient for the value estimate would indicate a stronger link to the rate of evidence accumulation. Both RL and WM value estimates were associated with CPP buildup rate (Figure 5). The association was stronger in lower set sizes for WM and in higher set sizes for RL (Table S1), corroborating previous behavioral, computational, and neural findings that WM contributes more to the decision signal when the number of items to remember is low and that the influence of RL increases with load.^21^^,^^24^^,^^26^ To ensure that these effects generalized across cohorts, we repeated the CPP slope analysis separately for the youth and pregnancy samples. Both groups showed a consistent divergence in the influence of RL and WM *Q* values on CPP slope across set sizes (see Figure S14), supporting the combined analysis.

## Discussion

In 510 participants, we investigated electrophysiological correlates of RL, WM, and choice dynamics in learning using the RLWM-LBA model.

Consistent with prior work using the softmax-based RLWM model, we identified distinct neural signatures associated with RL and WM processes. Specifically, we partially replicated previously documented temporal and topographic dissociations between RL and WM components.^24^^,^^26^ We also note, however, that we observed more widespread effects than previously reported, including earlier effects for set size. These partial discrepancies might be due to the considerably larger sample included in the current study, and to differences in the computational model: the RLWM-LBA jointly models RT and choice, which may alter the estimation of latent variables (e.g., *Q* values, drift rates) used to predict neural activity.

Further, we identified a CPP signal consistent with evidence accumulation processes previously described in perceptual and preference-based decision-making tasks.^15^^,^^16^^,^^29^ Our analysis revealed that CPP amplitude varied systematically with drift rate estimates from the RLWM-LBA model, such that lower drift rates were associated with prolonged and slower CPP buildup and lower peak amplitudes. These findings support the hypothesis that neural evidence accumulation processes represented in the CPP are engaged during decision-making in learning, where decisions reflect the internal uncertainty of learned action policies, rather than external sensory evidence. A recent study also found a CPP signal during internal sampling of evidence in which participants remembered the direction of a previously presented stimulus in a WM task.^35^

A recent study suggested that CPP effects can appear not because they are linked to evidence accumulation but due to the overlap of non-evidence-related activity linked to stimulus and response,^20^ but see O’Connell et al.^33^ Although early effects of increased activity for lower drift rates and slower RT disappear when applying the unfold method to deconvolve potentials associated with stimulus and response,^28^ effects closer to response remained, suggesting that the CPP response is linked to evidence-related processes.

At lower set sizes, we found weak or absent modulation of CPP slope and amplitude by drift rate.^22^ This pattern is consistent with theoretical and neural evidence suggesting that when WM contributes more strongly to behavior—as expected under low cognitive load—stimulus-response associations are acquired rapidly rather than incrementally. Consequently, trial-by-trial variability in decision evidence is reduced, leading to smaller differences between high- and low-evidence trials. In contrast, as set size increases and WM contributes less reliably to choice, learning relies more strongly on RL, which updates values gradually across trials. This leads to greater variability in accumulated evidence and stronger modulation of the CPP by drift rate. These findings align with a cooperative framework in which WM and RL jointly support learning, with RL contributing more to evidence accumulation under higher cognitive load.^24^^,^^26^

There were strong effects of drift rate in the peak amplitude of activity, such that higher evidence trials produced higher peak amplitudes (Figure 4). Similar amplitude modulations have been observed in both perceptual and preference-based decision-making tasks.^14^^,^^17^^,^^31^^,^^32^ One possible explanation is that decision boundaries dynamically collapse over time,^36^^,^^37^ or that an urgency signal accelerates the decision process by amplifying the decision variable as time elapses.^38^^,^^39^ Both mechanisms predict that when decision uncertainty is high, the system may commit to a choice with less accumulated evidence—resulting in lower thresholds and correspondingly lower CPP amplitude. These strategies have been shown to support near-optimal behavior under time constraints,^40^ and may help explain why lower drift trials are associated with lower CPP peaks. However, distinguishing between these alternatives was beyond the scope of the current study. Future work could investigate whether incorporating collapsing bounds or urgency mechanisms enhances the explanatory power of accumulation models in RL contexts.

We found that incorporating a trial-by-trial EEG-derived CPP buildup signal into the RLWM-LBA model improved model fit. This result demonstrates the utility of leveraging neural data to refine computational models of learning and decision dynamics.^2^^,^^31^^,^^34^^,^^41^^,^^42^^,^^43^ Importantly, our analyses extend prior findings by showing that trial-level fluctuations in neural accumulation signals not only reflect but also actively inform the dynamics of decision-making during learning. Future studies could test whether implementing neural measures into the RLWM-LBA model as we did here improves association of computational phenotypes to measures of development and mental health.^27^^,^^44^^,^^45^^,^^46^^,^^47^

Our results also contribute to the ongoing discussion regarding the interplay between RL and WM systems.^24^^,^^25^^,^^44^^,^^48^^,^^49^ We observed that the relative contributions of RL and WM to decision-related neural dynamics varied systematically with cognitive load, where differences in *Q* values were more associated with the CPP buildup in lower set sizes for WM and higher set sizes for RL, reinforcing the concept of a cooperative yet dynamic relationship between these cognitive processes.

In conclusion, by integrating EEG data with the RLWM-LBA modeling framework, our findings provide robust empirical support for a neural evidence accumulation mechanism underlying RL decisions. This extends our understanding of choice dynamics in learning contexts and offers valuable methodological insights for future research exploring the complex interactions between multiple cognitive systems during learning.

### Limitations of the study

While our findings suggest a neural evidence accumulation mechanism during RL, alternative interpretations warrant consideration.^20^ For instance, CPP signals might partly reflect action monitoring^50^ or decision confidence^51^ rather than pure accumulation per se. However, we emphasize that the identified CPP signal closely mirrors neural accumulation dynamics extensively validated in perceptual decision-making studies, where careful controls and targeted manipulations have systematically addressed and ruled out several alternative explanations.^16^^,^^18^^,^^29^^,^^33^ Thus, although we cannot exclude contributions from other cognitive processes, the strong similarity in the temporal dynamics, topographical distribution, and decision-related modulation of the CPP identified here with studies on perceptual decision-making lends support to our interpretation that the CPP reflects evidence accumulation processes during RL.

Some methodological considerations should be acknowledged. First, the current design did not include stimulus-onset jitter, potentially resulting in overlapping neural responses to stimulus presentation and evidence accumulation, complicating clear isolation of decision-related processes. Future studies employing jittered stimulus presentations could enhance the temporal specificity of CPP effects. Second, the model was suboptimal at capturing the evolution of RTs for incorrect responses. Although we note that only correct response trials were included in the EEG analyses, this issue might be mitigated by the implementation of collapsing boundaries or urgency signals, which can capture how decisions with high uncertainty can result in faster responses.^52^

## Resource availability

### Lead contact

Further information and requests for resources and reagents should be directed to and will be fulfilled by the lead contact, Mads L. Pedersen (m.l.pedersen@psykologi.uio.no).

### Materials availability

This study did not generate new reagents.

### Data and code availability

• The de-identified behavioral and EEG-derived summary data supporting the findings of this study are available upon reasonable request and subject to a data transfer agreement due to ethical and privacy restrictions.
• Analysis code has been deposited at the Open Science Framework (https://osf.io/y72kh/) and is publicly available as of the date of publication. The DOI and persistent link are listed in the key resources table.
• This study did not generate additional unique datasets or resources.

## Acknowledgments

This study was funded by research grants from the 10.13039/501100005416Research Council of Norway (249795, 273345, and 300767), the 10.13039/501100006095South-Eastern Norway Regional Health Authority (2018076, 2019101, and 2021040), the 10.13039/501100002347German Federal Ministry of Education and Research (grant no. 01ZX1904A), The European Union-funded Horizon Europe project “environMENTAL” (101057429), KG Jebsen Stiftelsen, the 10.13039/501100000781European Research Council under the European Union’s Horizon 2020 Research and innovation program (802998), and the Department of Psychology, 10.13039/501100005366University of Oslo. Data was handled inside Service for Sensitive Data (TSD), a platform owned by the University of Oslo, operated, and developed by the TSD service group at the University of Oslo IT-Department (USIT). Computations were also performed using resources provided by UNINETT Sigma2—the National Infrastructure for High Performance Computing and Data Storage in Norway (NS9666S). We thank Michael J. Frank for providing feedback on an earlier version of the manuscript.

## Author contributions

M.L.P., L.T.W., and T.M. conceived the study; M.L.P. implemented the RLWM-LBA model and wrote the manuscript; T.M. conducted EEG analyses; E.R.F. and L.T.W. contributed to interpretation; E.R.F. collected and curated EEG and behavioral data. All authors contributed to manuscript revision and approved the final version.

## Declaration of interests

The authors declare no competing interests.

## Declaration of generative AI and AI-assisted technologies in the writing process

During the preparation of this work, the author(s) used ChatGPT in order to improve the readability and language of the manuscript. After using this tool/service, the author(s) reviewed and edited the content as needed and take(s) full responsibility for the content of the published article.

## STAR★Methods

### Key resources table

**Table:** 

REAGENT or RESOURCE	SOURCE	IDENTIFIER
**Software and algorithms**		

MATLAB	https://www.mathworks.com/	2023a;RRID:SCR_001622
Python	https://www.python.org/	3.11;RRID:SCR_008394
R	https://www.r-project.org/	4.5.2;RRID:SCR_001905
Stan	https://mc-stan.org	2.35.0;RRID:SCR_018459
Unfold	https://www.unfoldtoolbox.org	1.2
cmdstan	https://mc-stan.org/docs/cmdstan-guide/	2.32.2
cmdstanr	https://mc-stan.org/cmdstanr/	0.9.0
Psychtoolbox	http://psychtoolbox.org	3;RRID:SCR_002881
EEGLAB	https://sccn.ucsd.edu/eeglab/	RRID:SCR_007292
RLWM-LBA model	https://osf.io/y72kh/files/xa2fw	
HARDWARE		
BioSemi ActiveTwo	https://www.biosemi.com/Products_ActiveTwo.htm	2
Cedrus	https://cedrus.com/rb_series/index.htm	RB-740

### Experimental model and study participant details

#### Participants

Participants were human subjects (Homo sapiens) recruited from two prospective cohorts within the ongoing Norwegian longitudinal BRAINMINT study.^53^^,^^54^^,^^55^ Data from the first session were used. The initial sample included 565 participants (214 from the youth cohort and 351 from the pregnancy cohort). After EEG preprocessing and quality control, 55 datasets (9.7%) were excluded due to excessive artifacts or insufficient remaining trials (<50% of trials), resulting in a final sample of 510 participants included in the single-trial EEG analyses. The youth cohort comprised 214 participants (71.1% female) aged 11–25 years (mean = 17.8, SD = 2.63). The pregnancy cohort comprised 351 women aged 22–43 years (mean = 32.1, SD = 3.47). Only the first session from each participant was included. Participants were recruited through social media, newspaper advertisements, and collaborations with other research projects. Inclusion criteria included Norwegian language proficiency and fulfillment of MRI safety criteria. Participants were not allocated to experimental groups; instead, within-subject variation in working memory load (set size) was analyzed. Sex was recorded at inclusion. Because the pregnancy cohort consisted exclusively of women, sex was not included as a primary analytical factor. The potential influence of sex or gender on behavioral and neural measures was not formally tested and therefore represents a limitation of the current study. Written informed consent was obtained prior to participation. For participants under 16 years of age, guardians provided informed consent. All procedures were approved by the Norwegian Regional Committees for Medical and Health Research Ethics (REK South East, ref. no: 2019/943 and 2019/345).

### Method details

#### Task

The RLWM task was presented using an MS-7816 computer (Micro-Star International Co., Ltd) with Windows 7 (Microsoft Corporation, 2009). Participants were comfortably seated 80 cm from a Benq xl2420t monitor (Benq Corp.) with a resolution of 1920 × 1080 and a refresh rate of 60 Hz. Stimuli were presented using the Psychophysics Toolbox (Version 3.0.11)^56^^,^^57^^,^^58^ running in MATLAB R2014a (The MathWorks, Inc.). Responses were given on a Cedrus RB-740 response pad (Cedrus Corporation). In total, the session consisted of five experimental tasks. The RLWM task was second in order, preceded by a 10 minute resting state EEG-recording.

The RLWM task (Figure 1A), originally described in^22^ is a deterministic stimulus-action association task in which participants are instructed to learn the correct action to stimuli. Here we used an adapted version described in.^27^

The task consisted of 11 blocks, within which participants learned to associate one of three button presses to between 2 and 5 stimuli. The stimuli in each block corresponded to a different category of stimuli (e.g. sports, fruits, places etc.). Each stimulus was associated with only one action, and feedback was deterministic. Participants were shown the entire set of stimuli at the beginning of each block to familiarize themselves with them. Each stimulus was presented within each block in a pseudo-randomized order until the correct response was given 4 out of the last 5 trials, with a minimum of 9 and a maximum of 15 presentations.

Participants were instructed that a given stimulus-response association did not inform about another stimulus-response association within a block, such that for a block with 3 stimuli it was not necessarily the case that each stimulus was associated with a separate action, thereby preventing participants to infer the correct action for a stimulus based on knowing the correct action for another stimulus.

Stimulus presentation was preceded by a presentation of a fixation cross for 0.5 s. Stimuli were presented for 7 s during which a response could be given for the entire presentation. A response ended the stimulus presentation, which was followed by 0.75 s feedback presentation (“Correct” for correct trials and “Try again!” for error trials).

#### RLWM-LBA model

The RLWM model was developed to account for the contribution of working memory in learning tasks typically modeled only by a reinforcement learning model. The RLWM consists of an RL and WM module that are combined to inform choices. See^22^^,^^24^^,^^27^ for model comparison identifying the RLWM as fitting observed choice better than simpler RL models. Here we use the recently developed Reinforcement Learning Working Memory-Linear Ballistic Accumulator (RLWM-LBA) model^25^ (Figures 1B and 1C), which was developed as an extension of the RLWM to capture the dynamics of choice during learning with the LBA model,^6^ which is a sequential sampling model that captures the choice and RT distributions of choices by assuming that choices are made as a race between alternative actions. The separate RL and WM modules are introduced before describing how they are combined to capture choice dynamics with the LBA model.

#### RL module

In the RL module learning is assumed to follow the delta learning rule,^59^ in which the expected reward *Q*_*RL*_(*s*_*t*_,*a*_*t*_) of a stimulus *s* and action *a* on trial *t* is updated based on the prediction error between observed and expected reward, proportional to the learning rate parameter *α*_*RL*_:

$$Q_{RL}\left(s_{t},a_{t}\right)=Q_{RL}\left(s_{t},a_{t}\right)+\alpha_{RL}\left(r_{t}-Q_{RL}\left(s_{t},a_{t}\right)\right)$$

To capture potential neglect of negative feedback, the learning rate is reduced in proportion to a bias parameter following negative prediction errors:

$$\alpha_{RL}=\left(1-bias\right)\alpha_{RL}$$

The bias parameter was bound to between 0 and 1. A bias parameter of 1 indicates total neglect of negative feedback, whereas a value of 0 indicates an equal rate of learning from positive and negative prediction errors.

Probability of choosing an action *a* for a given stimulus *s* is captured by the softmax choice rule, which scales expected rewards to sum to 1 according to the sensitivity parameter *β*:

$$P_{RL}\left(a|s\right)=\frac{e^{\left(\beta_{RL}Q_{RL}\left(s,a\right)\right)}}{\sum_{i}e^{\left(\beta_{RL}Q_{RL}\left(s,a_{i}\right)\right)}}$$

#### WM module

The WM module is designed as a temporary storage with perfect updating, but with limited capacity and decay. The updating of expected reward *Q*_*WM*_ in the WM module follows the delta learning rule but with a learning rate *α*_*WM*_ set to 1:

$$Q_{WM}\left(s_{t},a_{t}\right)=Q_{WM}\left(s_{t},a_{t}\right)+\alpha_{WM}\left(r_{t}-Q_{WM}\left(s_{t},a_{t}\right)\right)$$

The bias parameter *bias* is also calculated for *Q*_*WM*_ (not shown). Given the delay-sensitive nature of working memory, the RLWM includes a decay parameter which assumes expected reward *Q*_*WM*_ for each trial decays toward the initial value of *Q*_*WM*_ before having observed any outcomes (here set to be 1/*n*_*actions*_) according to the decay parameter *ϕ*:

$$Q_{WM}\left(s_{t},a_{t}\right)=Q_{WM}\left(s_{t},a_{t}\right)+\phi \left(\frac{1}{n_{actions}}-Q_{WM}\left(s_{t},a_{t}\right)\right)$$

In the RL module, the probability of choosing action *a* for stimulus *s* is captured with the softmax choice rule:

$$P_{WM}\left(a|s\right)=\frac{e^{\left(\beta_{WM}Q_{WM}\left(s,a\right)\right)}}{\sum_{i}e^{\left(\beta_{WM}Q_{WM}\left(s,a_{i}\right)\right)}}$$

Finally, the proportion of which the two modules inform choices is described by the mixing value *W*_*WM*_:

$$\pi \left(a|s\right)=W_{WM}P_{WM}\left(a|s\right)+\left(1-W_{WM}\right)P_{WM}\left(a|s\right)$$

Importantly, the value of *W*_*WM*_ depends on the capacity parameter *C* and propensity to use WM *ρ*:

$$W_{WM}=\rho (\min\left(1,\frac{C}{ns}\right)$$

Where *C* is a value between 2 and 5 (number of stimuli within a block) and *ρ* is a proportion (i.e. between 0 and 1) representing the individual propensity to use WM in selecting actions.

#### Choice dynamics

The standard RLWM model^22^ captures choice probabilities of internal value estimates through softmax transformation. In the RLWM-LBA model, choices and the RT of choice is captured with the LBA model (Figure 1C). The LBA model captures decision between N alternatives as a race in which the winning alternative is the one that first crosses the decision threshold *b*, which is the sum of starting point bias *A* and the relative threshold *k*. The decision process is assumed to start randomly within the range of 0 and the starting point bias *A*. The speed of evidence accumulation for each decision is captured by the latent variable drift rate *V*. Lastly, time spent on stimulus encoding and motor response is captured by the non-decision time parameter *τ*.

Driven by the discovery that choice latency continues to decrease after choice performance asymptotes, McDougle and Collins^25^ incorporated a Shannon entropy transformation^60^ which captures the relative uncertainty regarding the specificity of stimulus-response-mappings across each action *a* and stimuli *s*, prior to encoding the current trial’s stimulus.

$${\vec{\pi }}_{i}^{\mu }=\frac{1}{n_{s}}\;\sum_{i}^{n_{s}}\pi_{i,s}$$

High uncertainty is associated with longer RTs.^61^ Thus, if the correct action is the same across stimuli within a block, uncertainty is low and is associated with faster RTs, while if the correct action differs across stimuli, uncertainty is high and is associated with longer RT.

$$H_{\mathrm{prior}}=-\sum_{i=1}^{3}\frac{⇀\mu }{\pi_{i}}\log_{2}\left(\frac{⇀\mu }{\pi_{i}}\right)$$

To incorporate stimulus-response-mapping uncertainty into the drift rate computation, the combined action policy estimate *π* is divided by the entropy measure *H*_*prior*_. In addition, the softmax-transformed choice probabilities are scaled with a scaling parameter *η* to ensure value estimates are scaled to capture choice latency:

$$V_{a,t}=\eta \left(\frac{\pi_{i,t}}{H_{\mathrm{prior},t}}\right)$$

Finally, the likelihood of trial-by-trial choice and RT is estimated with the LBA probability density function using trial-by-trial estimates of internal uncertainties for each of the three response alternatives *V*_*a*,*t*_ together with LBA-parameters of starting point bias *A*, the relative threshold *k*, variability in drift rate *s* and non-decision time *τ*.

$$(\mathrm{Choice},\;\mathrm{RT})\;∼\;\mathrm{LBA}\left(k,A,V_{a,t},\tau \right)$$

#### Model analysis

We modeled performance in the RLWM task with a hierarchical Bayesian model in STAN^62^ via the cmdstanr package^63^ in R. A Bayesian framework allows for utilizing prior knowledge in the definition of prior distributions and insight into the uncertainty of estimates through the estimation of posterior distributions. A hierarchical approach estimates group and individual parameters simultaneously, which has been shown to improve parameter recovery for individual parameters, in particular with fewer data points.^64^

Due to the large sample size, we used the Variational Bayes method to approximate posterior distributions. The Variational Bayes method, in contrast to Markov Chain Monte Carlo (MCMC) sampling, does not guarantee a perfect approximation of the posterior distribution, and cannot be used to assess convergence, but is orders of magnitude faster than MCMC methods. Group priors were set to be in the range of previously reported estimates for each parameter. Weakly informative group priors were set to be in the typical range of estimates for each parameter. Priors for mean estimates of the parameters were as follows:

*μα* ∼ *normal*(−3,3)
*μbias* ∼ *normal*(−1,3)
*μϕ* ∼ *normal*(−1,3)
*μρ* ∼ *normal*(2,3)
*μC* ∼ *normal*(3,3)
*μk* ∼ *normal*(1,3)
*μA* ∼ *normal*(2,3)
*μη* ∼ *normal*(3,3)
*μτ* ∼ *normal*(-1,3)

Priors for standard deviations were all set to *exponential*(0.1). To constrain the parameters *α*, *ρ*, *ϕ*, *τ* and *bias* between 0 and 1, we approximated individual-level parameters using the normal CDF (via Phi_approx) for the group-level prior. Subject-level parameters were estimated as deviations from the mean group parameter, inspired by,^1^ in order to ensure exploration of parameter space for low between-subject variability. Priors for all subject-level parameters were set to *normal*(0,1).

We used the LBA probability density function and random generation function created for Stan to respectively calculate the likelihood of parameters given choice and RT and to generate predicted choice and RT for posterior predictive checks.^65^ Inferred latent variables directly calculated in the model were used as trial-by-trial predictors in the single trial EEG analyses.

#### Model comparison

Model comparison was performed using leave-one-out cross-validation (LOO), summarized by the expected log predictive density (ELPD).^66^ This approach estimates how well each model predicts unseen data and inherently penalizes model complexity, as overly flexible models are less likely to generalize well and thus obtain lower ELPD values. Differences in ELPD substantially larger than their associated standard errors are interpreted as strong evidence for improved generalization.

#### EEG

##### EEG data acquisition

EEG was recorded at a sampling rate of 2048 Hz using 64 active Ag/AgCl electrodes (placed in a cap in accordance with the 10/5 international system) with a BioSemi ActiveTwo system amplifier (BioSemi B.V., Amsterdam). This system minimizes common mode voltages by using a driven right leg electrode. Electrode offset was maintained between -20 and 20 mV. In addition to the 64 cap electrodes analyzed in the current study, we also recorded data from 8 external electrodes: two electrodes placed approximately 1 cm left to the left eye and right to the right eye measured horizontal eye movements (electrooculogram; EOG); two external electrodes placed on the left iliac crest and right clavicle measured the electrocardiogram, ECG); two electrodes were placed on the left and right abductor pollicis brevis measured electromyographic activity related to manual responses).

##### EEG preprocessing

Continuous EEG data was first high pass filtered at 0.1 Hz using the eeglab firfilt_new funtion with default parameters, before using the eeglab cleanline plugin to attenuate elecrical line noise at 50 Hz (as well as harmonics up to 1000 Hz). Next, the data was downsampled to 512 Hz, and bad scalp channels were identified using the eeglab clean rawdata function and rejected if their mean correlation with neighboring channels was below 0.8 or the line noise relative to its signal was above 4 standard deviations. Excluded channels were finally interpolated, before low-pass filtering the data at 40 Hz and extracting epochs ranging from -1.5 to 3 seconds relative to stimulus presentation. Epoched data was next decomposed using independent component analysis (ICA - picard algorithm), and resulting independent components were assigned probabilities of representing brain activity or common EEG artifacts using IClabel. Components assigned probabilities > .30 of belonging to the class “brain” and > .50 of belonging to the class “other” were retained for further analysis as these criteria had previously been found to identify most artifactual components. Next, we imported key single trial parameter estimates from the computational models (e.g., reinforcement learning Q values) into the epoched datasets and removed trials with not to be included in the analyses (e.g., error trials or the first presentation of a stimulus within a block), as well as any trials with amplitudes exceeding +/- 150 μV between 1 second prior to and 3 seconds after stimulus onset. Datasets with less than 50% of the original 468 trials remaining were excluded, leaving a total of 510 datasets for single trial EEG analysis (i.e. 55 of EEG datasets (9.7%) were excluded). Finally, we extracted epochs from –700 to 700 ms relative to stimulus onset (for stimulus-locked analyses) or relative to the response (for response-locked analyses).

##### Single trial EEG analysis

Single trial EEG analyses were conducted using linear modelling as implemented in the Unfold toolbox.^28^ In brief, EEG voltages at all electrodes and timepoints were fitted by general linear models, where the EEG signal is modelled as the sum of a set of independent variables/predictors, plus random noise.

For the stimulus-locked analyses we fitted models that included a fixed intercept (modelling the grand average ERP across all trials), as well as three predictors modeling trial-to-trial variability in set size (ranging from 2 to 5 stimulus-response associations), delay (i.e., the number of trials since the last correct performance of this specific stimulus-response association) and reinforcement learning Q value. All these trial-specific predictors were z-transformed prior to being included in the EEG-analyses.

For the response-locked analyses we fitted models that included a fixed intercept (modelling the grand average ERP across all trials, as well as one predictor modelling trial-to-trial variability in drift rate. As for the stimulus-locked analyses, this trial-specific predictor was z-transformed before being included in the EEG analyses.

Each single-trial analysis resulted in a set of beta-weight (for each predictor) and these single subject beta-weights were next aggregated at the group level, where we computed 20% trimmed mean ERPs (ensuring estimates robust against potential outlier values), and tested for statistical significance using trimmed mean t-tests. All resulting p-values were Bonferroni-corrected across electrodes (64), time-points (716) and estimated parameters (4 for stimulus-locked analyses, 2 for response-locked analyses), yielding a Bonferroni-corrected threshold of 2.73e-06.

#### Control analyses deconvolving stimulus and response-related effects

For the control analyses, checking if the main stimulus- and response-locked results were still reliable after explicitly modeling temporally overlapping responses and stimuli, we followed the standard analysis pipeline in the Unfold toolbox.^28^ Specifically, we first concatenated the artifact-pruned long epochs (-1500 to 3000ms relative to stimulus onset) to create an artifact-free EEG-dataset including sufficiently long stretches of continuous EEG around stimuli and responses to allow their associated ERP-signatures to be deconvolved. Next, we defined and fitted a design matrix that included 1) fixed intercepts (modelling the grand average ERP across all trials) for both the stimulus and the response, and 2) stimulus and response-related ERP effects modelled by trial-to-trial variability in the predictors derived from the computational models. For both stimuli and responses, we modelled the interval from -700 to 700 ms relative to stimuli/responses and used the default stick-function approach for this time-expansion. Each control analysis thus yielded twice as many beta-estimates as the main analyses (disentangling effects linked to stimuli and responses). As for the main analyses, all trial-specific predictors were z-transformed prior to being included in the control EEG-analyses.

### Quantification and statistical analysis

Behavioral and EEG data were analyzed using hierarchical Bayesian modeling and linear regression approaches. The RLWM-LBA model was fit to trial-level choice and RT data using variational inference in Stan. Model comparisons were conducted using expected log predictive density (ELPD) via leave-one-out cross-validation. Single-trial EEG data were analyzed using linear modeling with the Unfold toolbox, relating trial-wise regressors (e.g., RL/WM Q-values, drift rate) to ERP signals. Trial-wise CPP slope estimates were used in Bayesian regression models (brms) and incorporated into a neurally-informed RLWM-LBA model. Posterior correlations were examined to assess potential tradeoffs between RL and WM latent variables. All analyses were performed in R 4.5.2, MATLAB R2023a, and Python 3.11.
