# Supplementary material for: Neural decision dynamics underlying reinforcement learning and working memory
Source: iScience. 2026 Mar 27;29(5):115471. doi: 10.1016/j.isci.2026.115471 (PMC13092874; doi:10.1016/j.isci.2026.115471)
Supplement: Document S1. Figures S1–S14 [file mmc1.pdf]

iScience, Volume *29*

Supplemental information

Neural decision dynamics underlying reinforcement learning and working memory

Mads L. Pedersen, Erik R. Frogner, Lars T. Westlye, and Torgeir Moberget

Supplemental Information

Table S1. Posterior distributions of difference on the association of RL and WM quartiles on CPP slope.

Set size contrast	RL difference (95% HDI)	WM difference (95% HDI)
3-2	0.004 [0.002, 0.006]	-0.002 [-0.004, -0.001]
4-2	0.006 [0.004, 0.008]	-0.004 [-0.006, -0.002]
5-2	0.007 [0.005, 0.008]	-0.004 [-0.006, -0.003]
4-3	0.002 [0.001, 0.003]	-0.001 [-0.003, 0.000]
5-4	0.001 [-0.000, 0.002]	-0.001 [-0.002, 0.001]

CPP = centro-parietal positivity, HDI = highest density interval, RL = reinforcement learning, WM = working memory.

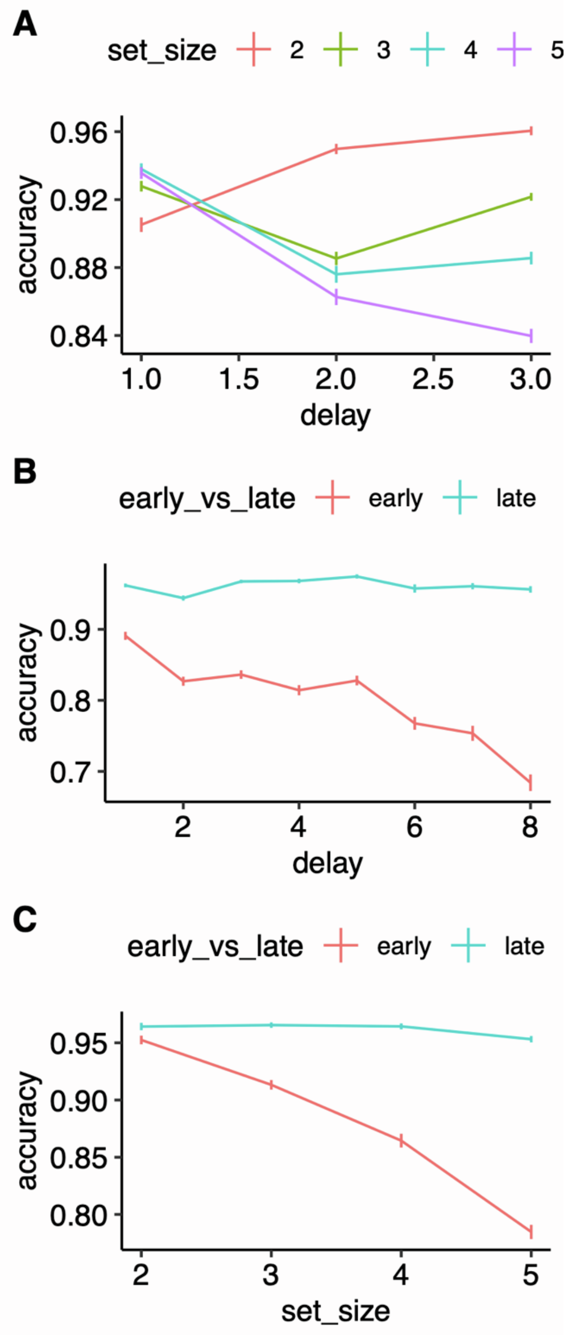

Figure S1. Behavioral results indicating involvement of working memory. (**A**) Accuracy decreases with delay in higher set sizes, suggesting the decay in working memory. (**B**) and (**C**) Declining effects of delay and set size on accuracy over trials point to a shift from WM to RL as learning stabilizes.

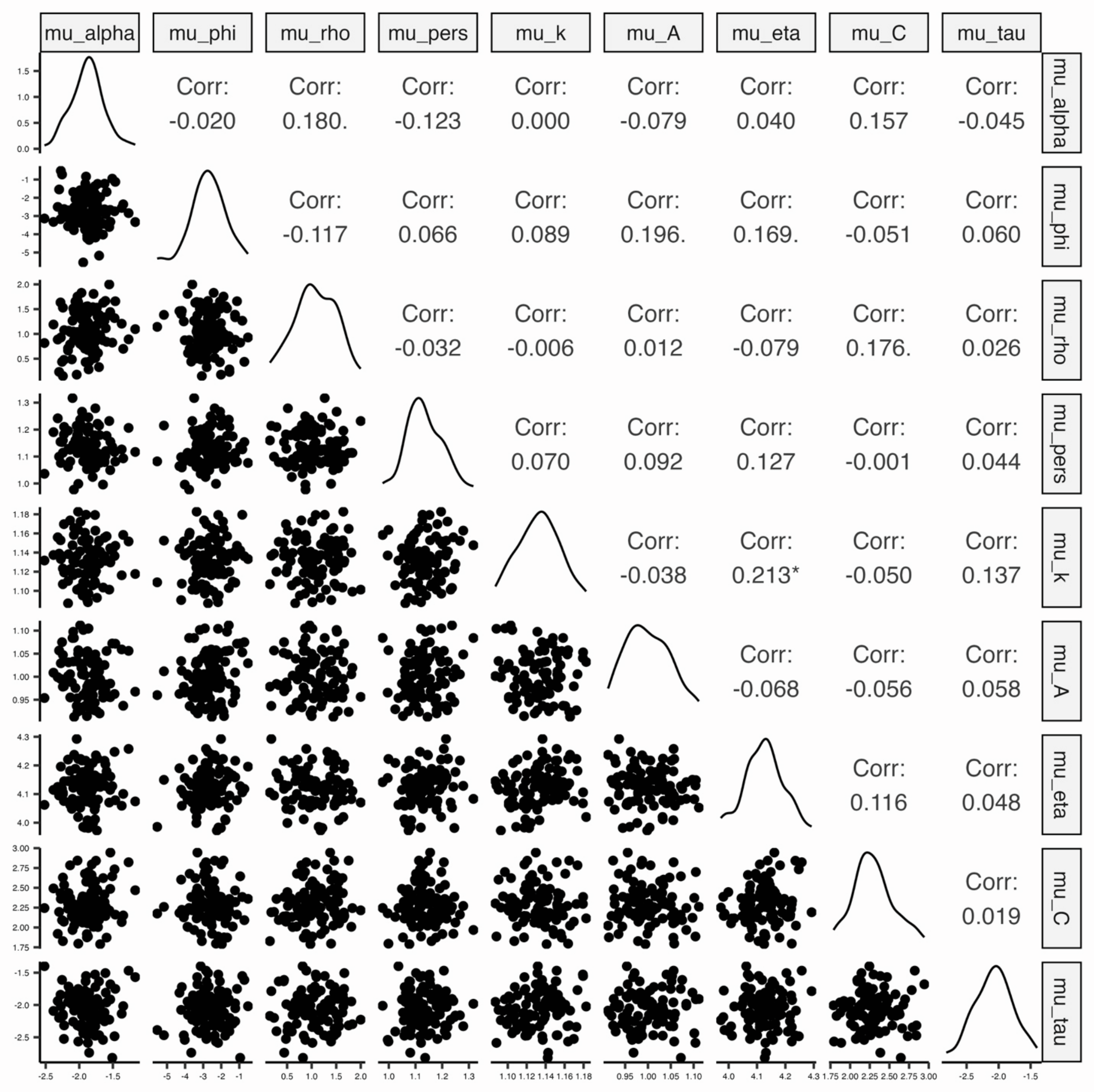

Figure S2. Scatterplot of group parameter estimates from RLWM-LBA model. A = starting point bias, k = relative decision threshold, eta = drift rate scaling parameter, phi = WM-decay, rho = WM-proportion, C = WM-capacity, pers = bias positive learning rate, tau = non-decision time, alpha = learning rate.

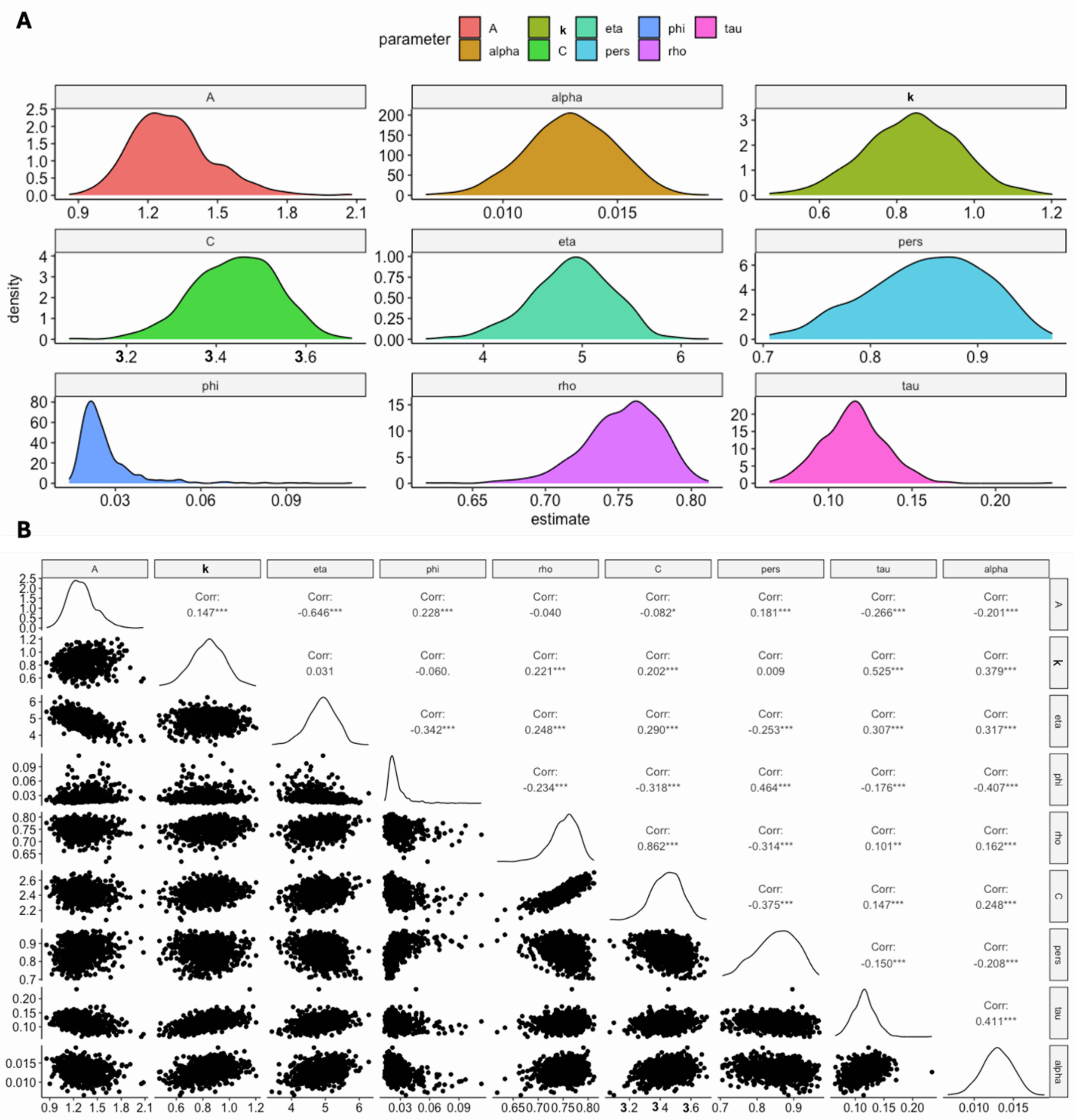

Figure S3. Parameter estimates from RLWM-LBA model. (**A**) Approximated posterior distributions and (**B**) scatterplots of individually estimated parameters. A = starting point bias, k = relative decision threshold, eta = drift rate scaling parameter, phi = WM-decay, rho = WM-proportion, C = WM-capacity, pers = bias positive learning rate, tau = non-decision time, alpha = learning rate.

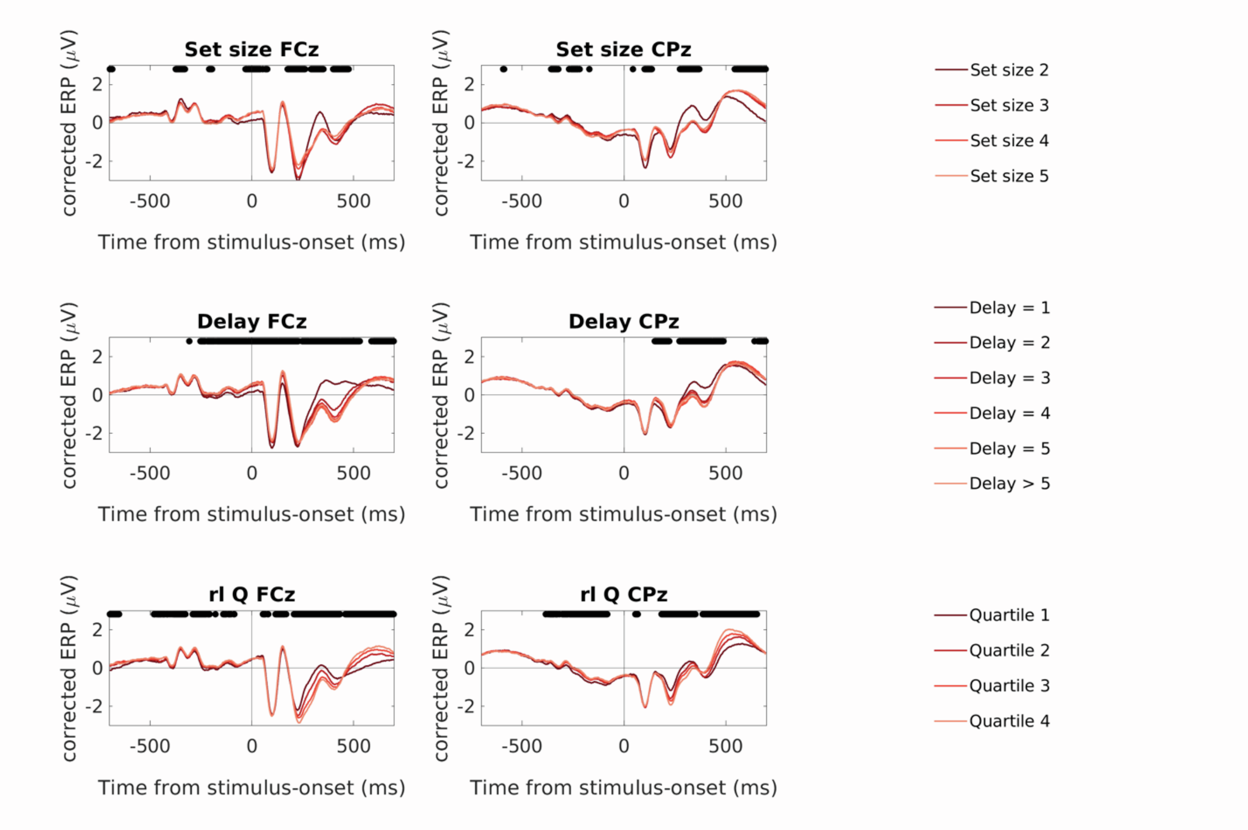

Figure S4: Corrected ERP effects of RL and WM predictors. Corrected ERPs showing the main effect of set size, delay and RL Q after removing effects of all other predictors. Black dots denote significant timepoints (after Bonferroni-correction across timepoints, electrodes and tested variables).

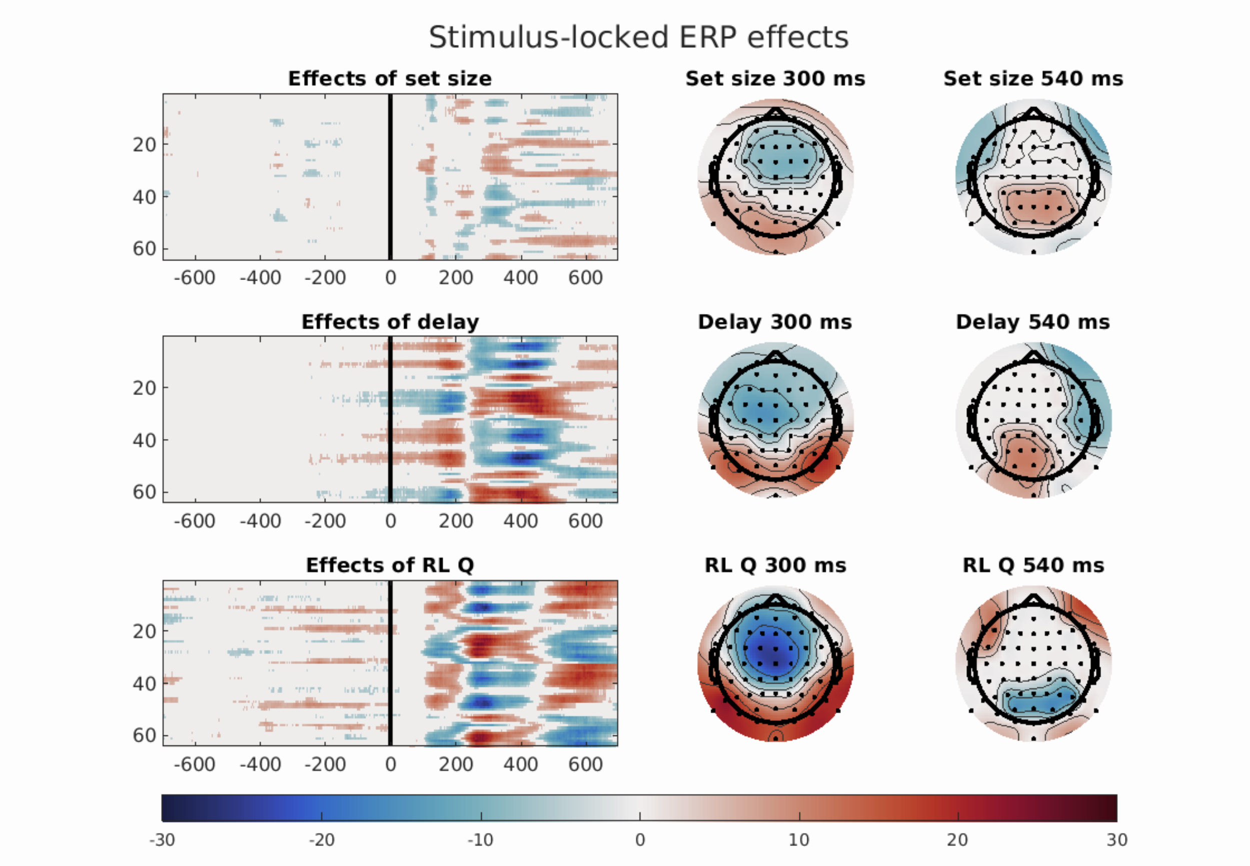

Figure S5. Stimulus-locked ERP effects of RL and WM predictors when accounting for overlapping response-related EEG activity. Colored tiles in the left panels denote significant t-values across timepoints (x-axis) and electrodes (y-axis). Right panels show scalp maps of effects of set size, delay and Q-RL-values at early (300 ms) and late (540 ms) after stimulus onset. Colored electrodes and time points show significant negative (blue) or positive (red) t-values (Bonferroni corrected across electrodes, timepoints and estimated model parameters), with the scale ranging from –30 to 30.

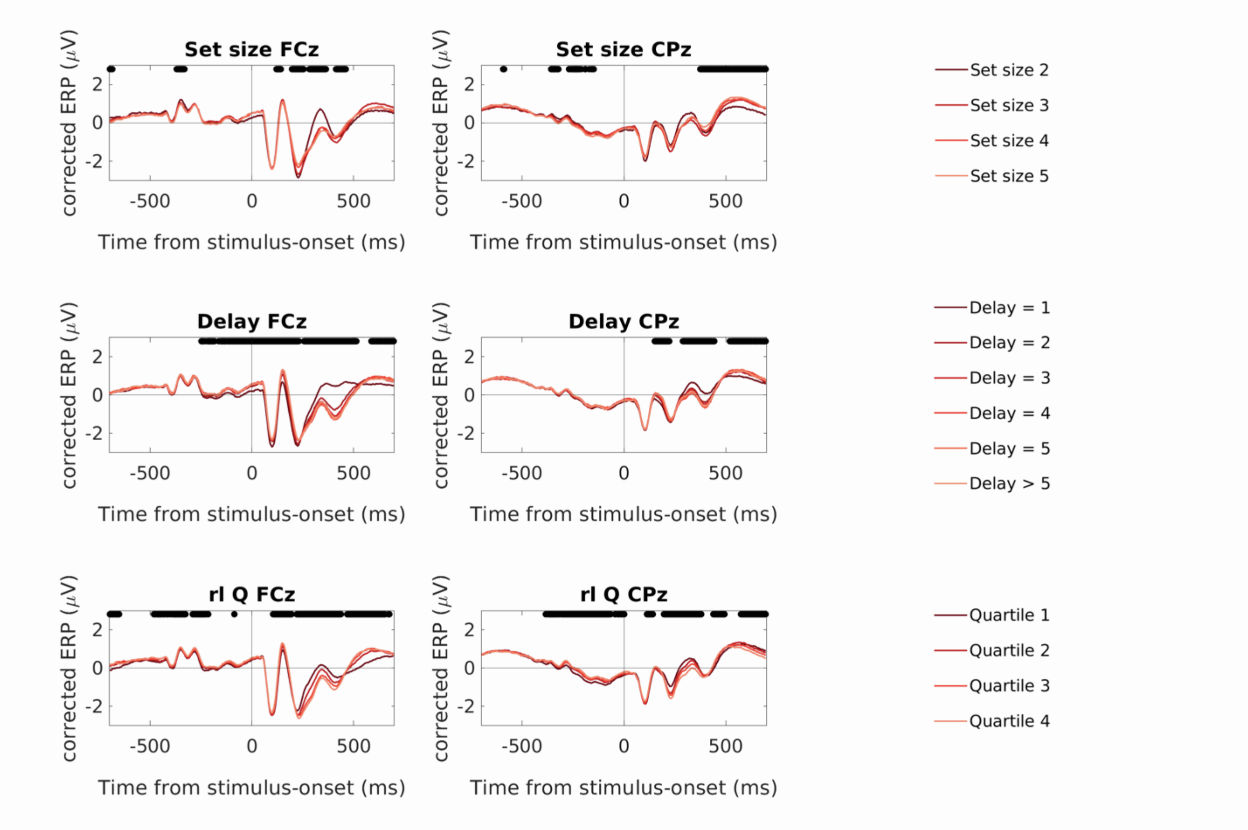

Figure S6. Stimulus-locked corrected ERPs when accounting for overlapping response-related EEG activity in the control analyses. Corrected ERPs showing the main effect of each predictor after removing effects of all other predictors. Black dots denote significant timepoints (after Bonferroni-correction across timepoints, electrodes and tested variables).

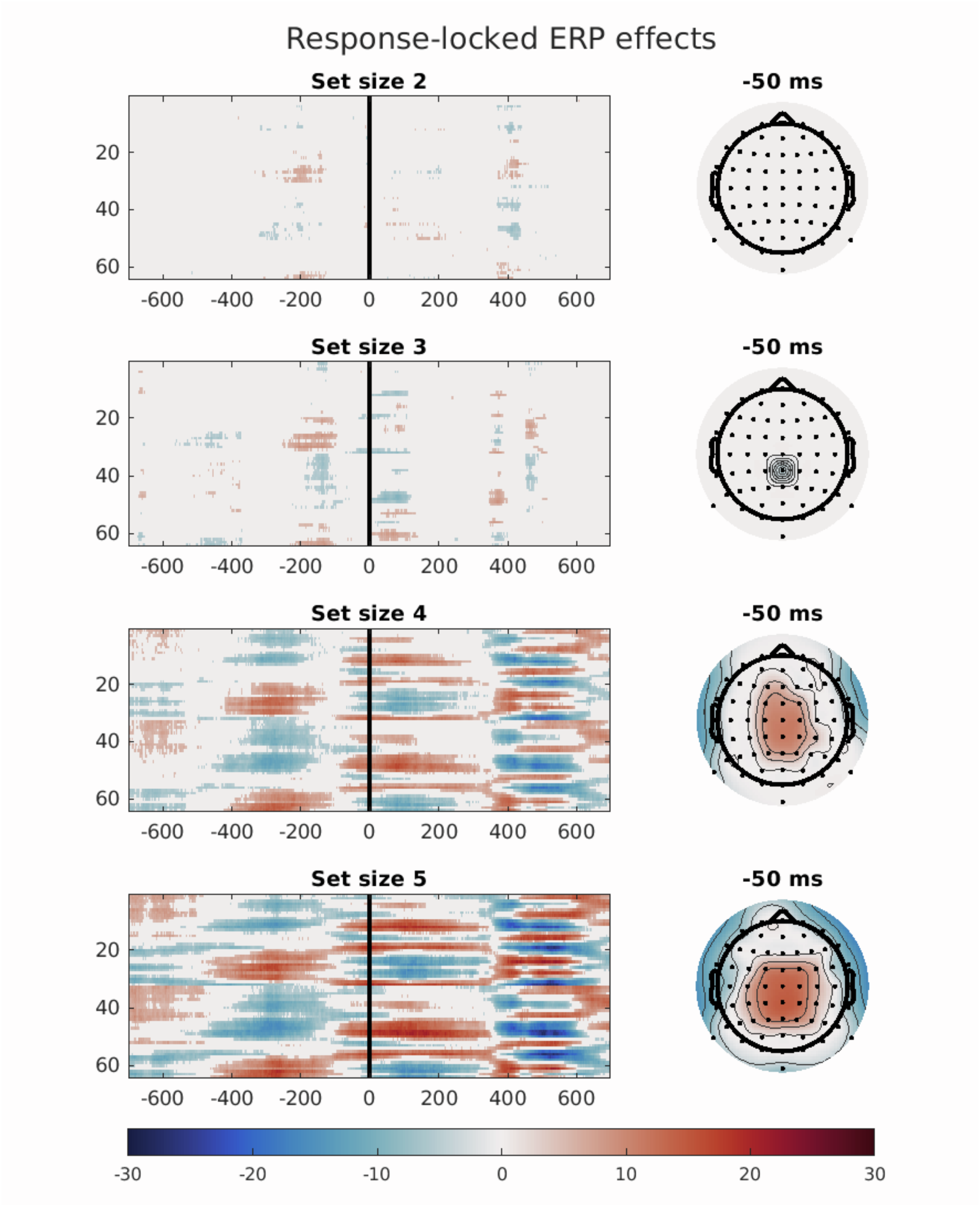

Figure S7. Response-locked ERP effects of drift rate. Left panels show ERP effects of drift rate across electrodes (y-axis) and timepoints (x-axis) for the four different set size conditions. Colored tiles in the left panels denote significant t-values across timepoints (x-axis) and electrodes (y-axis). Right panels show scalp topographies of t-values just prior to the response. All results have been thresholded at $p < 0.05$ after Bonferroni-correction across timepoints, electrodes and tested variables. White regions signify non-significant effects.

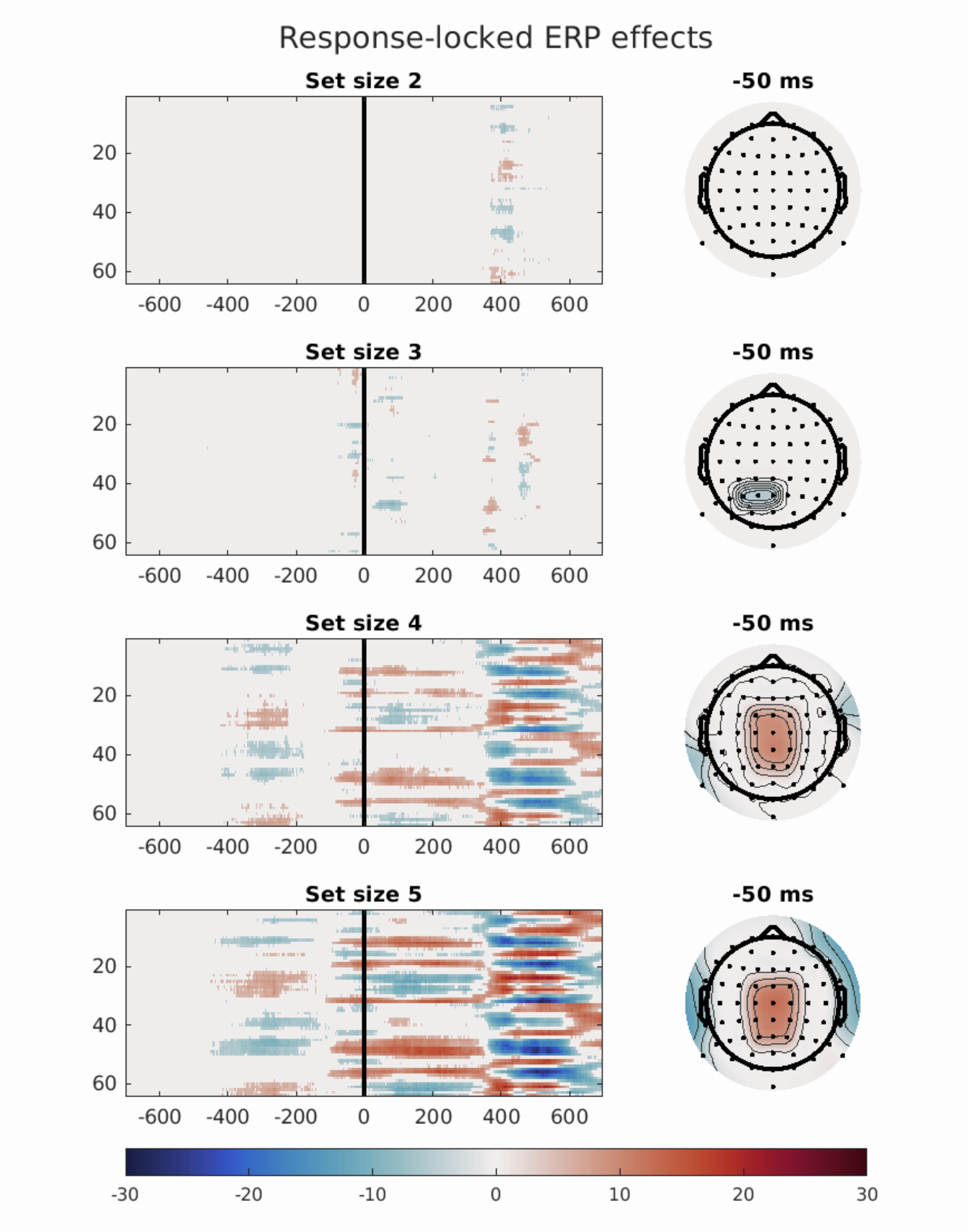

Figure S8. Response-locked ERP effects of drift rate when accounting for overlapping stimulus-related EEG activity. Colored tiles in the left panels denote significant t-values across timepoints (x-axis) and electrodes (y-axis). Right panels show scalp topographies of t-values just prior to the response. All results have been thresholded at $p < 0.05$ after Bonferroni-

correction across timepoints, electrodes and tested variables. White regions signify non-significant effects.

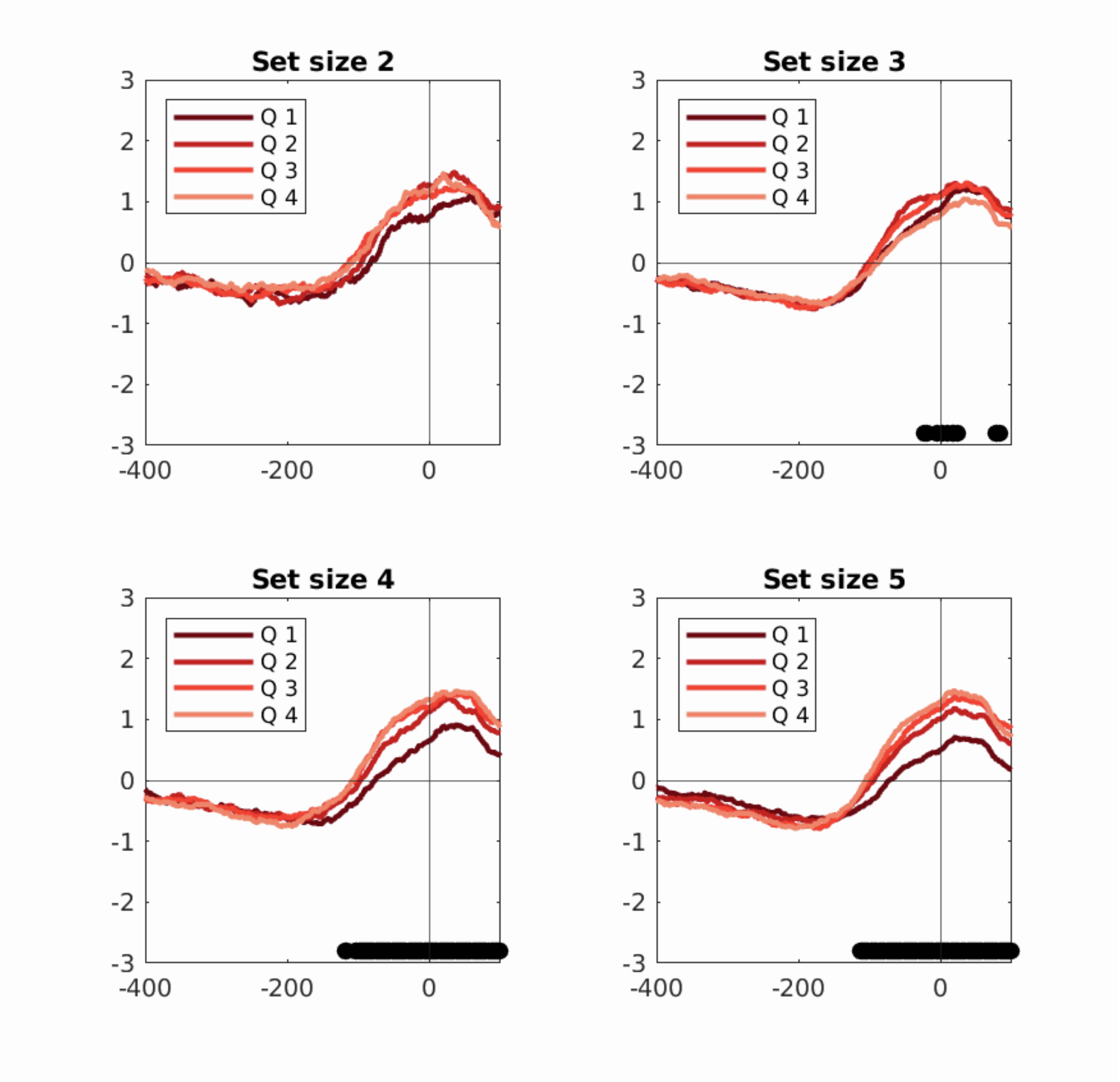

Figure S9. Response-locked ERP effects of drift rate quartiles on electrode CPz when accounting for overlapping stimulus-related EEG activity. Inferred drift rates were divided into quartiles (Q) for each subject and set size condition. Q1 represents the trials with the least and Q4 the trials with the most evidence for the chosen (correct) option. The x-axis shows time (in ms) relative to the response, while the y-axis shows ERP amplitudes (in microvolts). Black dots denote significant timepoints (after Bonferroni-correction across timepoints, electrodes and tested variables).

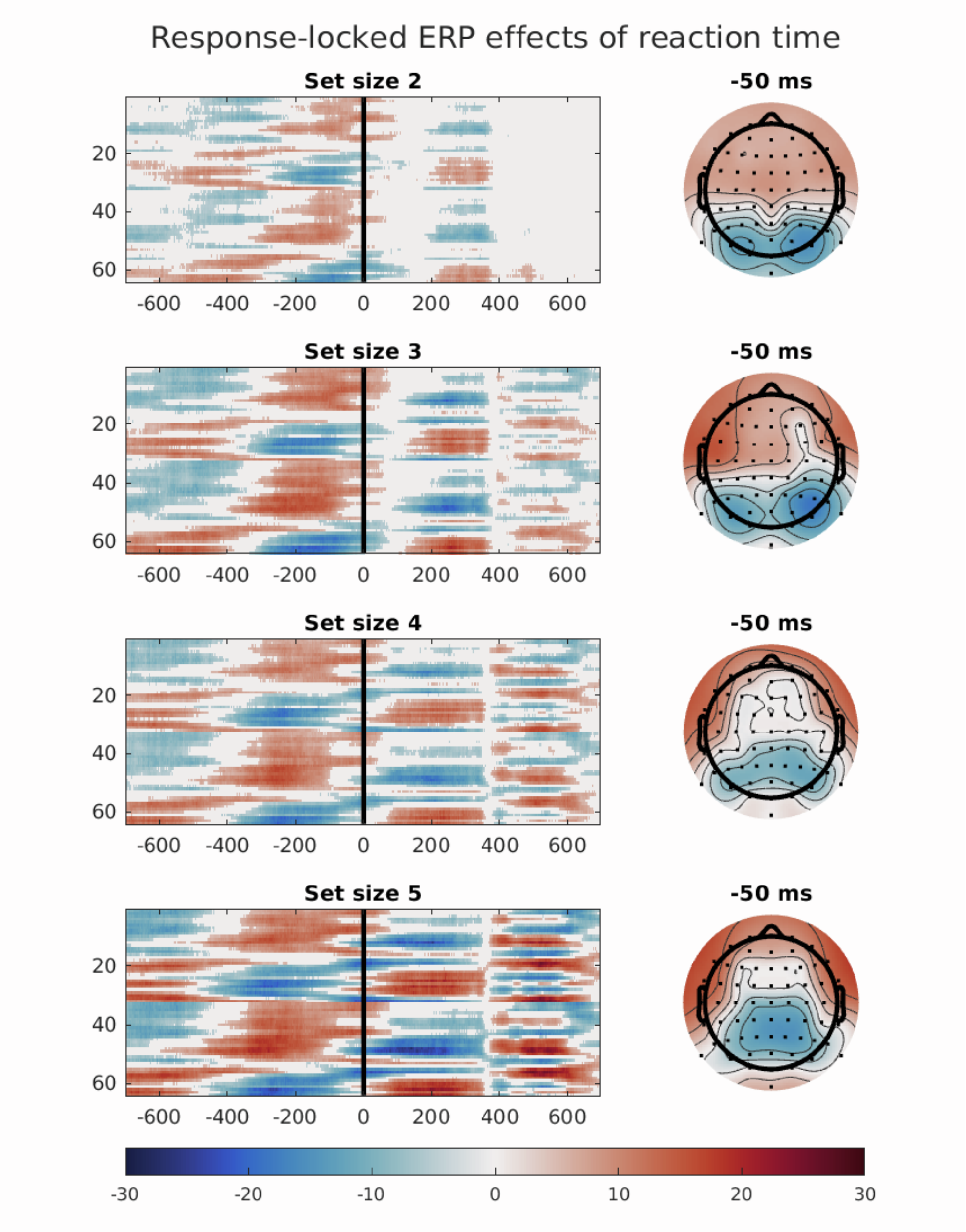

Figure S10. **Response-locked ERP effects of response time.** Colored tiles in the left panels denote significant t-values across timepoints (x-axis) and electrodes (y-axis). Right panels show scalp topographies of t-values just prior to the response. All results have been thresholded at $p < 0.05$ after Bonferroni-correction across timepoints, electrodes and tested variables. White regions signify non-significant effects.

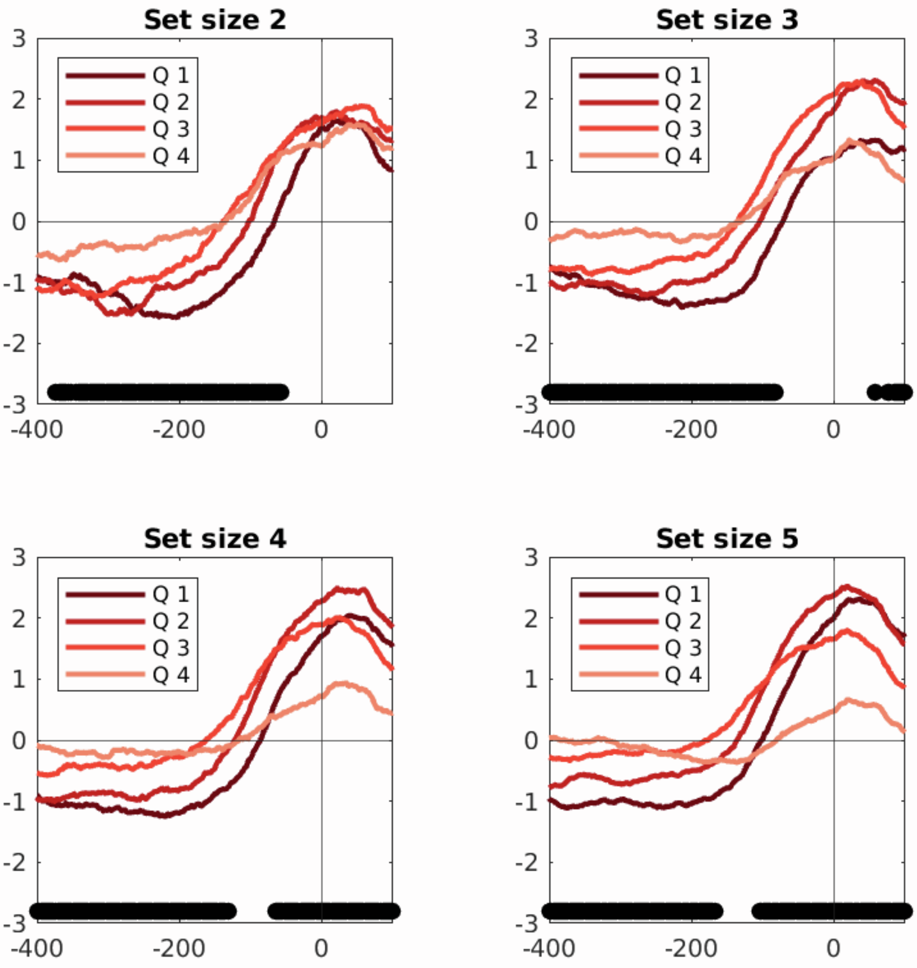

Figure S11. Response-locked ERP effects of response time quartiles on electrode CPz. Response times were divided into quartiles (Q) for each subject and set size condition. Q1 represents the fastest trials and Q4 the slowest for the chosen (correct) option. The x-axis shows time (in ms) relative to the response, while the y-axis show ERP amplitude (in microvolts). Black dots denote significant timepoints (after Bonferroni-correction across timepoints, electrodes and tested variables).

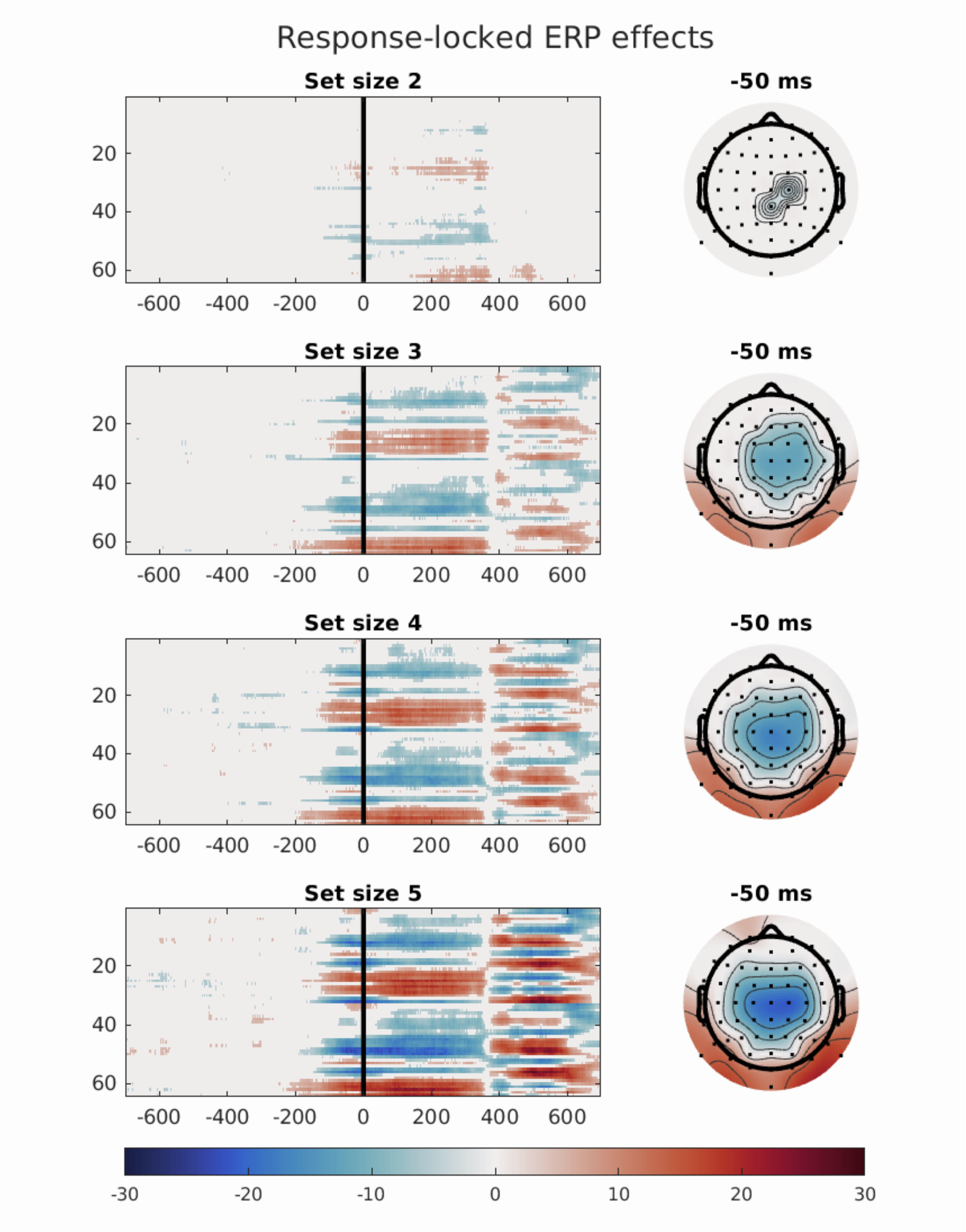

Figure S12. Response-locked ERP effects of response time across all electrodes and timepoints when accounting for overlapping stimulus-related EEG activity. Colored tiles in the left panels denote significant t-values across timepoints (x-axis) and electrodes (y-axis). Right panels show scalp topographies of t-values just prior to the response. All results have been thresholded at $p < 0.05$ after Bonferroni-correction across timepoints, electrodes and tested variables. White regions signify non-significant effects.

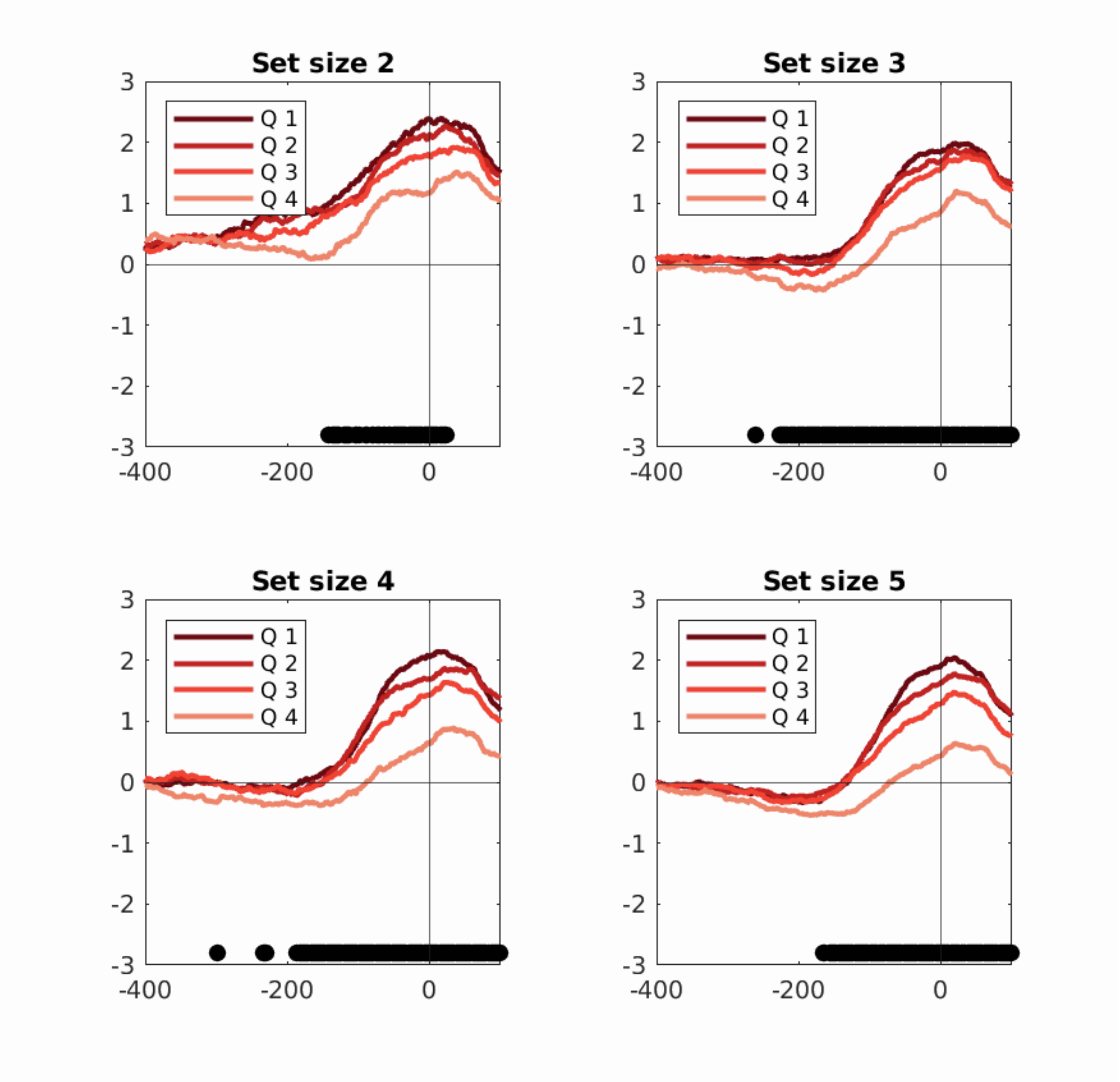

Figure S13. Response-locked ERP effects of response time quartiles on electrode CPz when accounting for overlapping stimulus-related EEG activity. Response times were divided into quartiles (Q) for each subject and set size condition. Q1 represents the fastest trials and Q4 the slowest for the chosen (correct) option. The x-axis shows time (in ms) relative to the response, while the y-axis shows ERP amplitude (in microvolts). Black dots denote significant timepoints (after Bonferroni-correction across timepoints, electrodes and tested variables).

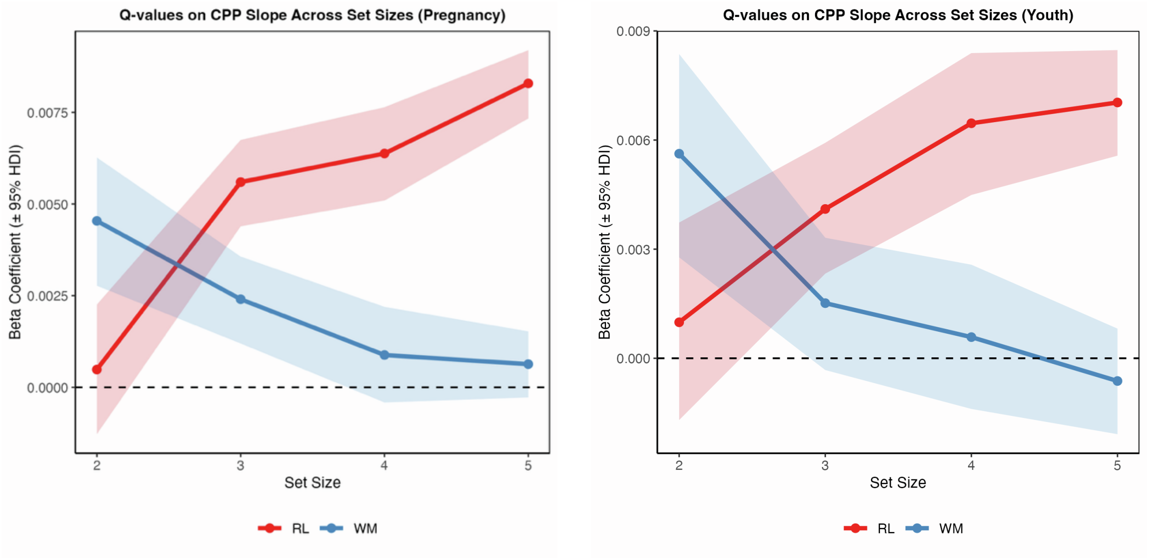

Figure S14. Associations of Q-RL (red) and Q-WM (blue) quartiles on CPP slope across set sizes split by pregnancy (left) and youth cohorts (right). Beta coefficients with 95 % highest density interval (HDI). The associations increased with set size for RL and decreased for WM, in line with assumptions of increased (decreased) influence of RL (WM) on choice with load.
